# Analysis of aggregation profile of glucagon using SEC-HPLC and FFF-MALS methods

**DOI:** 10.1371/journal.pone.0304086

**Published:** 2024-05-21

**Authors:** Zhongli Bao, Ya-Chi Cheng, Mary Ziping Luo, Jack Yongfeng Zhang

**Affiliations:** Amphastar Pharmaceuticals, Inc., Rancho Cucamonga, California, California, United States of America; GLA University, INDIA

## Abstract

Recently, the first generic glucagon for injection was approved for the treatment of severe hypoglycemia. Unlike its brand name recombinant glucagon, the generic glucagon is synthetic. Since glucagon has a high propensity to form aggregates in solution, it is essential to assess the aggregation profile of the synthetic glucagon compared to the recombinant glucagon. In this study, two robust separation methods, size-exclusion chromatography (SEC-HPLC) and field-flow fractionation coupled with a multi-angle light scattering detector (FFF-MALS), were employed to characterize generic and brand glucagon aggregation in six lots (three newly released, three expired). The presence of aggregation in samples was determined from the generated chromatograms and analyzed. The study showed that both products have comparable aggregation profiles. The SEC-HPLC demonstrated that in both glucagon versions, the expired lots had a higher percentage of dimers than the newly released lots, but even at expiration, the amount was negligible (∼0.1%). The FFF-MALS method did not detect any dimers or higher molecular weight aggregates. Further evaluation of the detection limit found that FFF-MALS was unable to detect aggregates at amounts lower than 0.5% of total glucagon. The negligible amounts of dimer detected in the generic and brand glucagon indicate that both versions are physically stable and are not prone to aggregation under clinically relevant conditions.

## Introduction

Protein or peptide aggregation is a concerning issue encountered in nearly all stages of drug development [1, 2]. The occurrence of aggregation reduces physical stability and functionality of the peptide or protein in question, which not only may lead to a loss in activity but can also create critical problems such as toxicity and immunogenicity [1]. Many factors can compromise the physical stability of therapeutic protein/peptides being developed including ionic strength, concentration, pH, and temperatures [1–4]. Therefore, the development of essential therapeutic protein/peptides such as glucagon, can be challenging and difficult due to their high tendency for aggregation.

Glucagon, a 29-amino acid peptide hormone, is an important therapeutic agent with many uses including the emergency treatment of severe hypoglycemia, where an individual’s blood sugar drops to a level that can cause confusion or unconsciousness [5]. However, glucagon has poor solubility and is prone to aggregation. For instance, glucagon can rapidly fibrillate, forming aggregates in solution [6]. As a result, pharmaceutical preparations of glucagon such as GlucaGen^®^ HypoKit^®^ (Novo Nordisk, Princeton, NJ) or Glucagon™ (Eli Lilly and Company, Indianapolis, IN)) are formulated as lyophilized powder to be reconstituted with diluent for immediate use [7]. Due to the complex nature of glucagon, there has been no approved generic version of this product for 20 years. In fact, glucagon had been included on the FDA’s list of off-patent, off-exclusivity drug products which encourages the development of a generic version.

Recently (Dec 2020), a generic version of glucagon (Amphastar Pharmaceuticals Inc., Rancho Cucamonga, CA) was finally developed and approved by the FDA [8]. The generic glucagon, like Eli Lily’s version, is also supplied as a lyophilized powder to be reconstituted to a concentration of 1 mg/mL with an aqueous pH 2.0 diluent. However, unlike the reference-listed drug (Eli Lilly’s) of glucagon, which is of recombinant DNA (rDNA) origin, the approved generic is a synthetic peptide product manufactured using a synthesis method. This led to the interest in studying the aggregation profile of the synthetic glucagon compared to the recombinant glucagon.

This paper presents the aggregation profile results of a generic and brand name glucagon. Size-exclusion chromatography (SEC) and field-flow fractionation (FFF) were employed as separation and characterization techniques to assess glucagon aggregation. The details and principles for each method are summarized below.

## Material and methods

### Principles of size-exclusion chromatography

SEC-HPLC is a robust method that is widely used for detailed characterization of therapeutic proteins and is considered a powerful technique for the quantification of protein dimers, trimers and oligomers [9–11]. The main advantage of this technique is the mild elution conditions that allow for the characterization of the protein/peptide with minimal impact on the conformation structure and local environment [9]. In summary, SEC separates molecules based on their size. The SEC column consists of spherical beads with a pre-determined pore size, through which molecules diffuse based on their molecular weight (MW). Larger molecules are excluded from most pores and flow through the column quicker with a lower retention time, while smaller molecules will enter the pores and elute last. Consequently, molecules separate based on their size as they pass through the column and are eluted in order of decreasing MW.

In SEC, the size-based separation allows the construction of a calibration curve based on proteins or polymers of known MW. By plotting log*M* vs. the elution (retention) volume, a third order polynomial is obtained with a linear region providing the highest resolution and MW accuracy [12]. The calibration curve can then be used to estimate the MW of an unknown analyte. In this study, the calibration curve is used to identify monomers and dimers in each glucagon sample.

### Principles of FFF-MALS

Field-Flow Fractionation (FFF) is a unique separation method that uses a force field application to separate and characterize samples over a broad range of sizes (1 nm ‐ 100 μm) [13–15]. The advantage of FFF resides in its essential tunability, i.e. by simply adjusting the flow rate, a single channel can be used to separate and analyze complex samples comprising molecules, particles and emulsions with superb resolution. Asymmetrical flow FFF (AF4) is a one-phase chromatography technique and is the most popular type of FFF. Briefly, carrier fluid is pumped through the inlet end exhibiting a laminar flow profile. A cross-flow is induced perpendicular to the channel flow, which exits the channel through the bottom wall fitted with semi-porous (ultrafiltration) membrane. The cross flow acts as a force field, concentrating the sample against the bottom wall. The combination of the two forces applied eventually results in the separation of the sample components according to their respective diffusion coefficient (i.e. their hydrodynamic radius or molar mass, respectively). Smaller particles diffuse higher into the channel than larger particles and experience faster lateral velocity due to the parabolic profile of the channel flow. In this way, samples are fractioned such that smaller particles in the sample will elute out first.

The FFF is coupled with a multi-angle light scattering detector (MALS) instrument together with refractive index (RI) and UV detectors for comprehensive characterization of the sample (size, molar mass, concentration, etc.). The MALS detector applies a light beam on the sample causing scattered light. The online static light scattering, refractive index, and UV measurements are collected at multiple scattering angles [13]. Based on the Rayleigh-Gans-Debye theory, the intensity of light scattered by a molecule measured by a TREOS II MALS detector, is directly proportional to the molar mass [16, 17].

### Sample preparation

The generic glucagon (synthetic) was provided by Amphastar Pharmaceuticals, Inc. Recombinant glucagon (Eli Lilly’s Glucagon™) was purchased commercially and used as the reference listed drug (RLD). In this study, the generic glucagon is referred to as AMP-glucagon and the RLD is referred to as ELI-glucagon. In both the SEC-HPLC and FFF-MALS studies, six lots of AMP-glucagon were analyzed and compared with six lots of the ELI-glucagon, of which three lots were recently released (new) and three were expired lots (old). All samples were freshly prepared and tested immediately after reconstitution. All laboratory chemicals used were analytical grade or higher. Standards for SEC experiments including aprotinin, angiotensin, and myoglobin were purchased from Sigma (St. Louis, MO). Bovine serum albumin (BSA) from Wyatt’s (WTC, Santa Barbara, CA) was used as the standard for FFF-MALS experiment.

### SEC-HPLC testing procedures

The standard diluent was prepared by combining 22 mL of Mobile Phase A (0.1% TFA) and 18 mL of Mobile Phase B (acetonitrile) in a 200 mL volumetric flask diluted with double distilled water (DDW). For the standard stock solution, 1.5 mL of diluent was added to 3 mg of aprotinin, angiotensin, myoglobin, and insulin. Standards were then prepared by adding 0.3 mL of diluent to 0.3 mL of each stock solution. Each glucagon sample was reconstituted with the included diluent and tested immediately after reconstitution.

SEC separation was carried out on an Agilent HPLC System with UV detector (Agilent Technologies, Santa Clara, CA). A Phenomenex Yarra 3 μm SEC-2000 column (300 × 7.8 mm; Phenomenex^®^, Torrance, CA) was employed at ambient temperature. Sample injection volume was 10 μL at a flow rate of 1.000 mL/min. The HPLC running time was 30 minutes with 0.1% TFA and acetonitrile used as mobile phase A and B, respectively. Table 1 summarizes the SEC-HPLC system conditions.

**Table 1:** SEC-HPLC system conditions.

Item	Description
HPLC System	Agilent HPLC with UV Detector
Column	Phenomenex Yarra 3 μm SEC-2000, 300 x 7.8 mm
Wavelength	214 nm
Mobile Phase Composition	A: 55% B: 45%, Isocratic
Flow Rate	1.000 mL/min
Column Temperature	Ambient
Injection Volume	10 μL
Run Time	30 minutes

The SEC-HPLC was calibrated with the following protein standards: angiotensin (1,297 Da), glucagon (3,482 Da), insulin (5,808 Da), aprotinin (6,500 Da), and myoglobin (17,800 Da). Using the GPC software, a third order polynomial regression was produced to fit the standard calibration.

Based on the calibration curve, the dimer and monomer are identified in each sample and the percentage of dimer was calculated according to the following equation:

$$\%\text{Dimer}=\frac{\text{Dimer}\;\text{Response}}{\text{Dimer}\;\text{Response}+\text{Monomer}\;\text{Response}}$$

The percentage of dimer for the generic glucagon was compared to that of the RLD, and their equivalence was evaluated based on the following equation.

$$\text{Equivalence}\;\text{Evaluation}\;\text{Criteria}\;(\text{EEC})\;\text{Upper}\;\text{Limit}=R_{\max}\;\cdot \;(1+\eta )$$

where *R*_max_ is the maximum (overall extreme) of the results for the RLD lots, i.e. the highest value for the tested characterization parameters; *η* is the allowed percentage range. Since glucagon dimers are considered impurities, the lower limit was not established due to it being a favorable outcome. In other words, only the upper limit was analyzed.

### FFF-MALS testing procedures

For glucagon sample preparation, 1.0 mL of the 0.01 N HCl (0.1 μm filtered), was added to each unit of drug product to reconstitute the lyophilized cake to obtain 1 mg/mL of glucagon. The injection volume of the glucagon sample was 100 μL. For positive control, one lot of 2 mg/mL bovine serum albumin (BSA) was diluted to 0.2 mg/mL such that the amount (25 μL) injected is equal to 5% of glucagon sample loaded. The BSA standard was injected at the beginning and the end of the sequence. BSA is a globular protein with a monomer molecular weight of 66.5 kDa that is known to form well defined oligomers in solution, and therefore represents a good choice as a standard in the characterization of protein studies [15, 18].

The FFF experiments were carried out using Eclipse^®^ AF4™ systems coupled with MALS from Wyatt Technology Corporation (WTC, Santa Barbara, CA). For FFF, a short channel with a spacer of 350 μm and 1 kDA PES membrane (Synder Filtration, CA) was used with a detector flow rate of 0.5 mL/min. The system testing conditions are summarized in Table 2. The average molar masses of glucagon samples were calculated using Wyatt ASTRA software with RID and 90° light scattering (LS2) signal. The calculated weighted-average molar masses were compared to the theoretical values for glucagon (3483 Da) and BSA (66.5 kDa) and the percentage of deviation was obtained.

**Table 2:** Summary of FFF-MALS system conditions.

Mobile Phase	0.01 N HCl (0.1 μm filtered)				
Time Table	**Mode**	**Duration (min)**	**Cross Flow Start (mL/min)**	**Cross Flow End (mL/min)**	**Flow Profile**
	Focus	1.0	1.50	1.50	Constant
	Focus Inject	4.0	1.50	-	Constant
	Elution	10.0	5.50	5.50	Constant
	Elution	5.0	5.50	0.00	Linear
	Elution	5.0	0.00	0.00	Constant
	Elution Inject	4.0	0.00	0.00	Constant
	Elution	1.0	0.00	0.00	Constant
Injection Volume	100 μL (for glucagon samples), 25 μL (for BSA sample)				
FFF Information	Device: Short Channel (SC) Membrane: PES 1 kDa, Synder Spacer Height: 350 μm Detector Flow: 0.5 mL/min				
Quality Control	BSA standard 2mg/mL (Wyatt). Inject 25 μL at the beginning and the end of the sequence.				
Samples to be Tested	Three lots of AMP-glucagon on release (within expiry) Three lots of AMP-glucagon at the end of proposed shelf life Three lots of ELI-glucagon product (within expiry) Three lots of ELI-glucagon product at the end of proposed shelf life				
Data Collection	RID, LS (light scattering at 3 angles), UV@214nm				

## Results

### Presence of dimer by SEC-HPLC

AMP-glucagon had an overall lower percentage of dimer (MW of ∼ 6800 Da) compared to that of the ELI-glucagon (Table 3). For the recently released lots (within expiry), dimer was not detected in all the AMP-glucagon samples, whereas a small amount (0.040%) was found in the ELI-glucagon. For expired lots, the dimer in the AMP-glucagon was also found to be comparable or lower to that of the ELI-glucagon (0.086% vs 0.125% respectively). The higher amount of dimer found in the expired lots was negligible. There were no higher molecular weight aggregates found in the AMP-glucagon and ELI-glucagon.

**Table 3:** SEC-HPLC test results of AMP-glucagon and ELI-glucagon.

Time Shelf Life	Products	Lot No.	Monomer MW	Dimer MW	% Dimer by SEC-HPLC	Mean of Dimer, %
Recently Released	AMP-Glucagon	102017	3648	ND	0.000%	0.000%
		102017A	3652	ND	0.000%	
		102017B	3632	ND	0.000%	
	ELI-Glucagon	C734350C	3662	6881	0.040%	0.040%
		C699511C	3641	6870	0.042%	
		C753564A	3650	6917	0.038%	
End of Shelf Life (expired)	AMP-Glucagon	021914	3659	6785	0.062%	0.086%
		021914A	3648	6596	0.080%	
		021914B	3665	6803	0.115%	
	ELI-Glucagon	C502399D	3635	6775	0.133%	0.125%
		C505143A	3638	6757	0.097%	
		C464099D	3659	6779	0.144%	

ND = Not Detected

**Fig 1:**
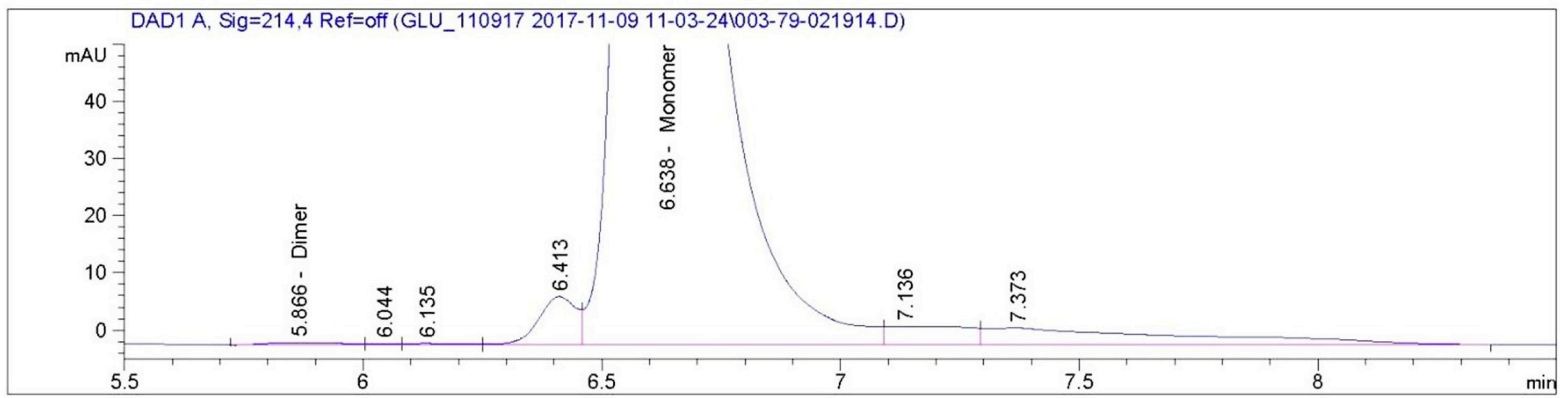
A representative SEC-HPLC chromatogram for expired AMP-glucagon: A dimer peak was observed at approximately 5.8 minutes.

**Fig 2:**
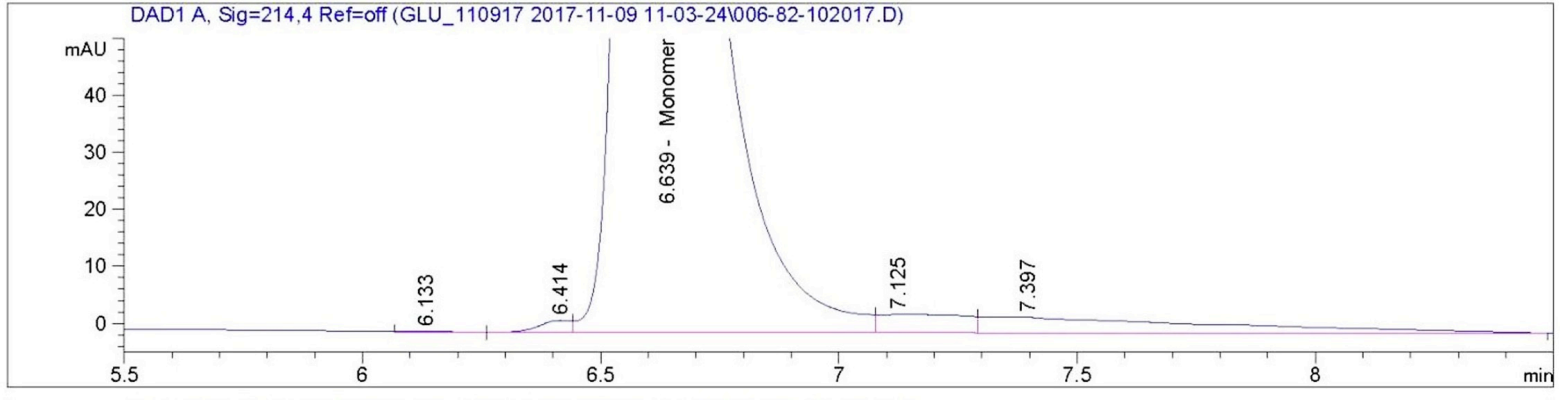
A representative SEC-HPLC chromatogram for recently released AMP- glucagon: No dimer peaks were detected.

**Fig 3:**
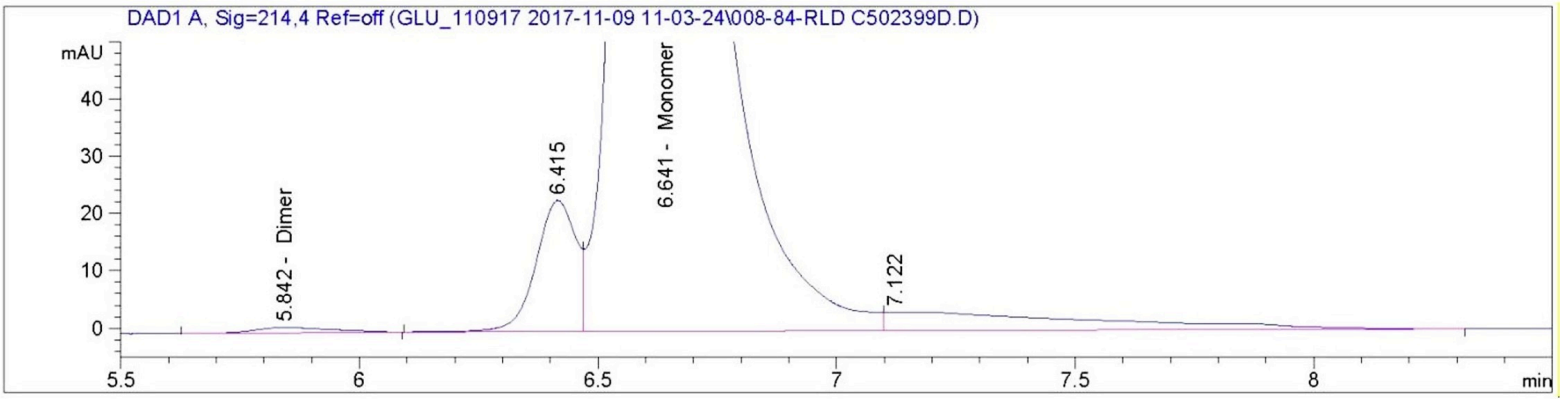
A representative SEC-HPLC chromatogram for expired ELI-glucagon: A dimer peak was observed at approximately 5.8 minutes.

**Fig 4:**
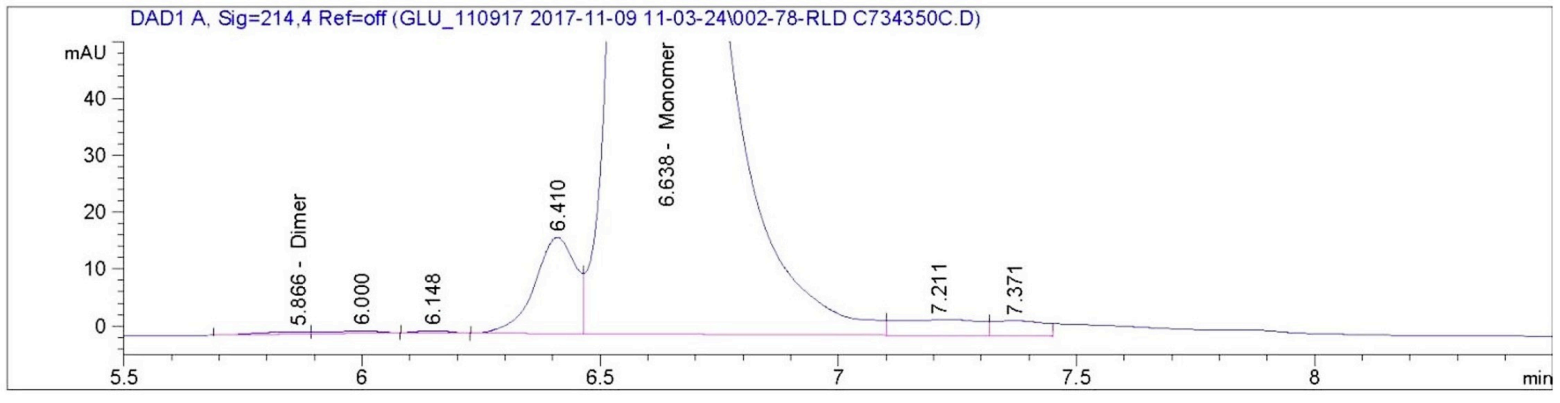
A representative SEC-HPLC chromatogram for non-expired ELI-glucagon: A dimer peak was observed at approximately 5.8 minutes.

The size exclusion chromatogram shows that glucagon monomers have a retention time of approximately 6.6 minutes in all of the AMP-glucagon and ELI-glucagon samples. Figs 1–4 show a representative SEC-HPLC chromatogram for one recently released lot and one expired lot of AMP-glucagon and ELI-glucagon, respectively. The SEC-HPLC chromatograms for all study lots are provided as a supplementary file (S1 Appendix). A dimer peak was observed at around 5.8 minutes for the expired AMP-glucagon lot (Fig 1), but none was detected in the recently released lot (Fig 2). For ELI-glucagon, a dimer peak was observed in both the recently released and expired lots at a similar retention time (5.8 minutes) to that of the AMP-glucagon (Figs 3 and 4).

An equivalence evaluation of the AMP-glucagon versus ELI-glucagon both at recently released and at expiration was conducted. From the signal to noise ratio, the limit of quantification (LOQ) was found to be around 0.1%. However, the dimer detection range for ELI-glucagon was highly varied with R_max_ being 0.042% for the recently released lot (which is way below the LOQ), and 0.144% (which is above the LOQ) for the expired lot (Table 4). Since the variation was found to be high at the dimer detecting range for ELI-Glucagon, for simplicity, the allowed percentage range (η) of dimer content was determined to be 25% for both recently released and expired lots. Therefore, the equivalence evaluation criteria (EEC) upper limit was defined as 1.25%*R_*max*_. The results of the EEC are summarized in Table 4, which shows the percentage of dimer of AMP-Glucagon products at release and at expiration; all met the EEC limits, indicating that AMP-Glucagon is equivalent to ELI-Glucagon for aggregation profile.

**Table 4:** Equivalence evaluation of aggregation of AMP-glucagon vs. ELI-glucagon By SEC-HPLC.

Type of Evaluation	Establishment of EEC for Aggregation Tested by SEC-HPLC		% Dimer	Meet EEC?
AMP- vs ELI-Glucagon at Release	Extreme of ELI-Glucagon (n = 3)	*R*_*max*_	0.042%	-
	EEC at Release*	Upper Limit	0.053%	-
	AMP-Glucagon Lot No.	102017	0.00%	✓
		102017A	0.00%	✓
		102017B	0.00%	✓
AMP- vs ELI-Glucagon at Expiration	Extreme of ELI-Glucagon (n = 3)	*R*_*max*_	0.144%	-
	EEC at Expiration*	Upper Limit	0.180%	-
	AMP-Glucagon Lot No.	21914	0.062	✓
		021914A	0.080%	✓
		021914B	0.115%	✓

*EEC = ELI-Glucagon Extreme + 25%, i.e. Upper Limit = 1.25*R_*max*_

### Characterization of glucagon aggregates by FFF-MALS aggregation profile comparison

**Fig 5:**
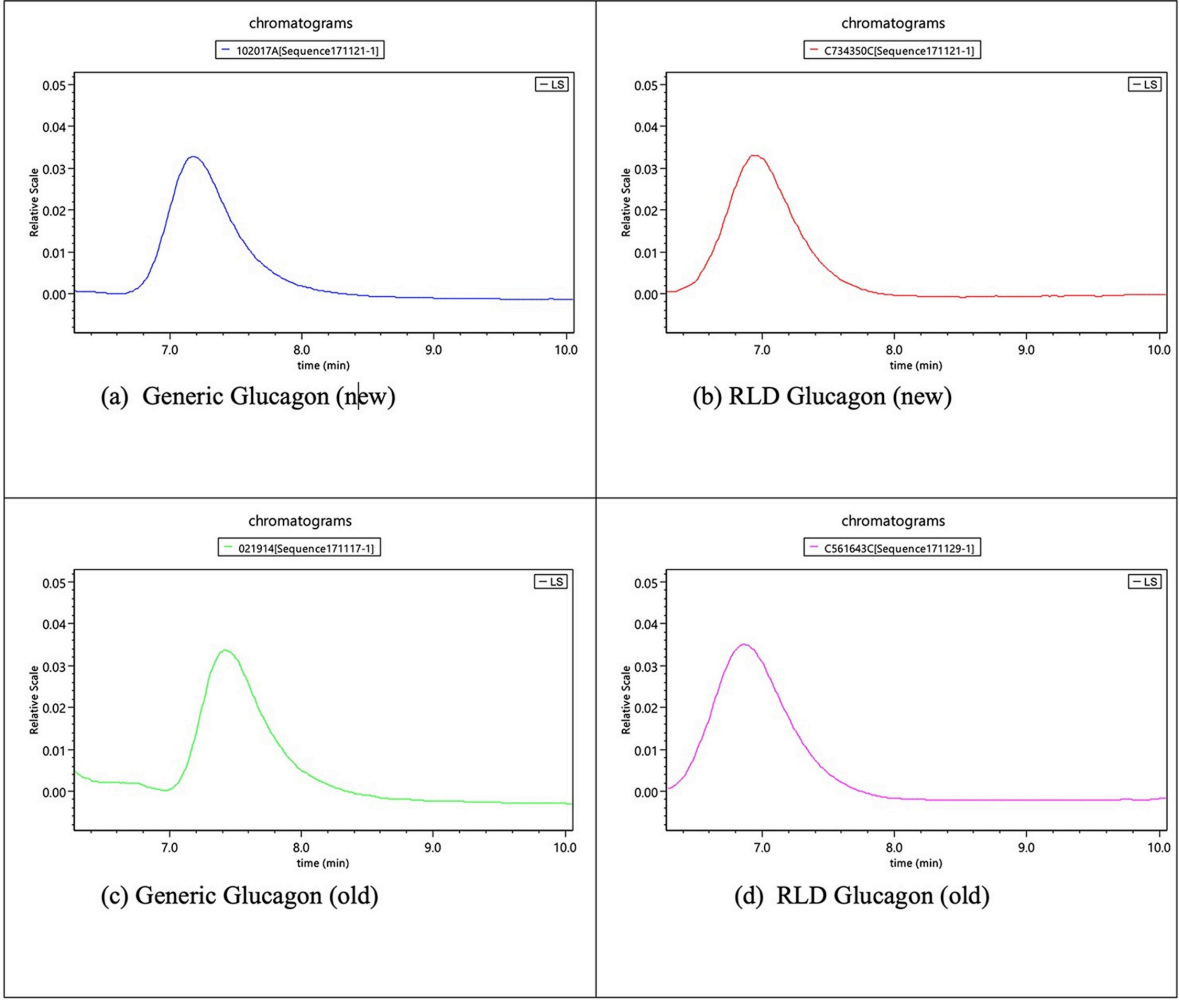
Typical chromatograms of LS2 signal for glucagon samples: No peak differences were observed between new (a, b) and old (c, d) lots in the two glucagon products.

The typical chromatograms of the LS2 (90° light scattering) for AMP-glucagon and ELI-glucagon are shown in Fig 5. No differences in terms of peaks were observed in all chromatograms between the two product groups. For all samples tested (new and old), only a single main peak, representing the glucagon monomer can be observed.

There is no aggregated glucagon detected by FFF-MALS in all glucagon samples. Theoretically, any detectable amounts of dimer or higher molecular aggregates in the sample should appear as a separate peak to the right of the main glucagon (monomer) peak. However, as shown in Fig 5, no such peaks were found in the chromatograms.

### Molar mass comparison

**Fig 6:**
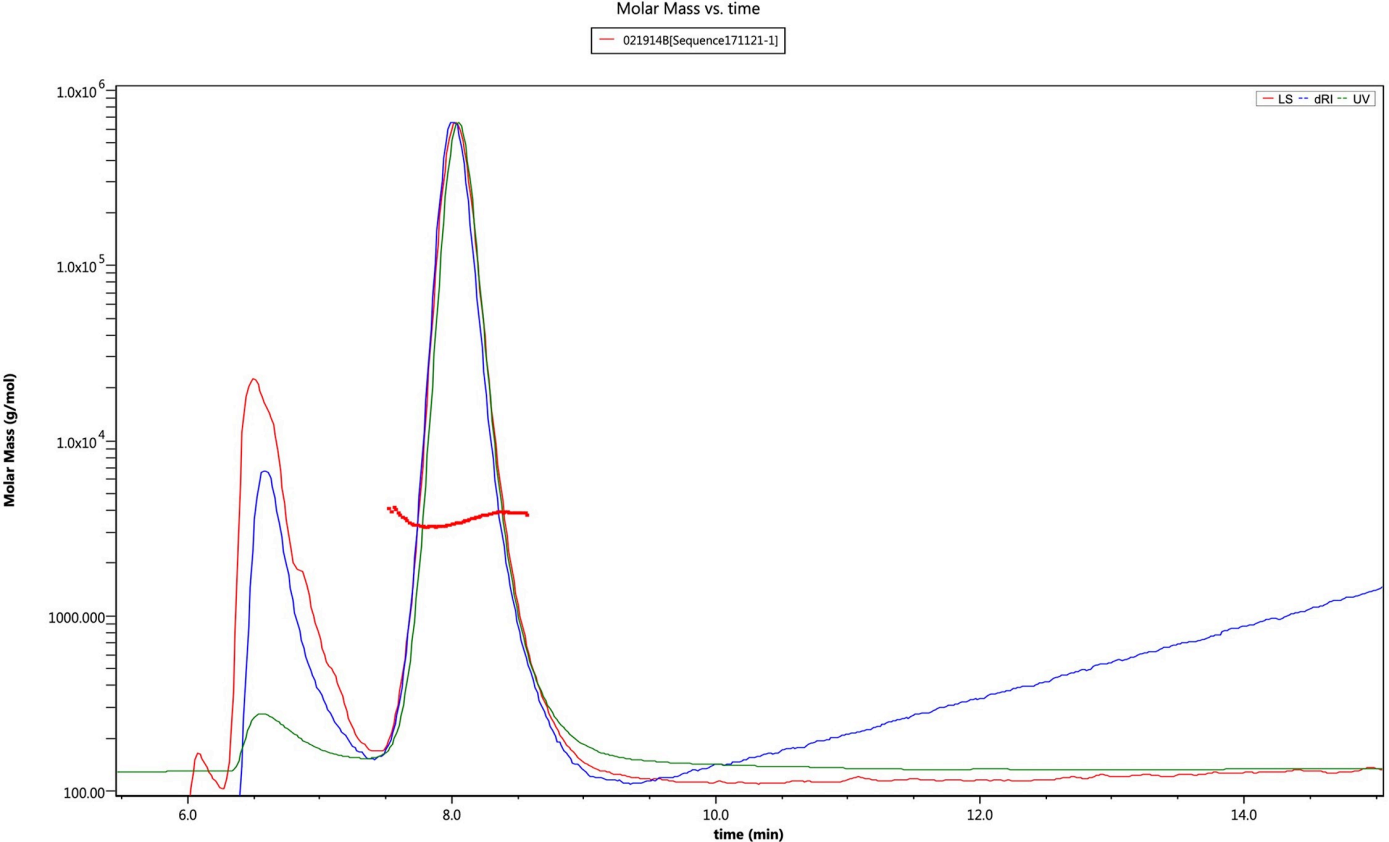
A Representative FFF chromatogram for AMP-glucagon: The molecular weight of glucagon was approximately 3,504 Da.

**Fig 7:**
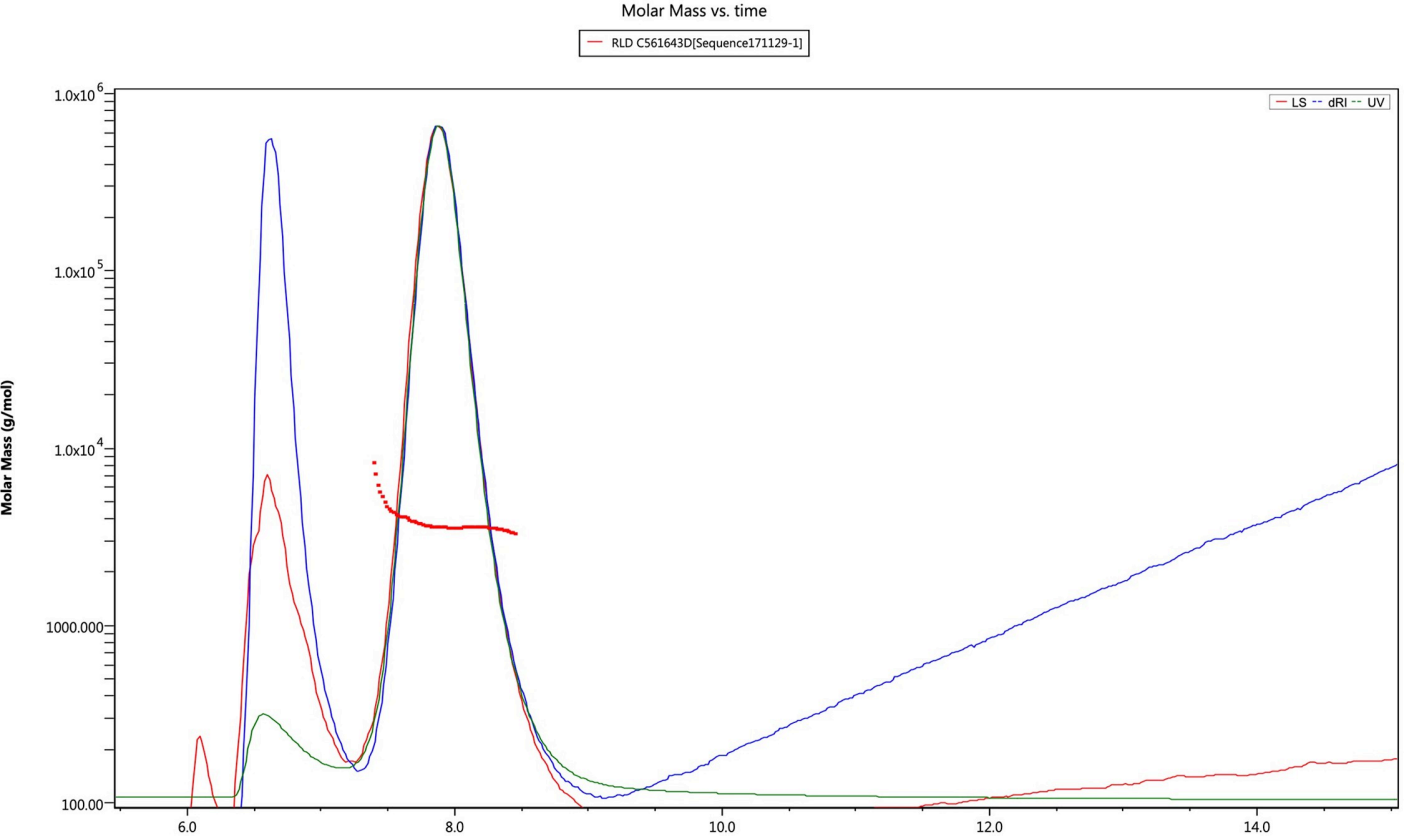
A representative FFF chromatogram for ELI-glucagon. The molecular weight of glucagon was approximately 3,720 Da.

The weighted-average molar masses were calculated using the Wyatt ASTRA software with data collected from the RID and LS2 signals. The molar mass results are summarized in Table 5 and two representative chromatograms are provided in Figs 6 and 7. The FFF chromatograms for all study lots are provided as a supplementary file (S2 Appendix). As shown in Table 5, the calculated weighted-average molar masses between AMP-glucagon and ELI-glucagon were found to be in good agreement. For the AMP-glucagon samples, the mean MW of the new lots was 3,612 Da and the old was 3,614 Da. The mean MW in the ELI-glucagon was similar, with the new lots having a mean MW of 3,636 Da, and the old having a mean MW of 3,622 Da. Compared to the theoretical MW of glucagon (3,483 Da), the mean MW of all glucagon samples had only a slight deviation of approximately 4%.

**Table 5:** Glucagon samples’ weighted-average molar masses by FFF/UV-MALS.

Time (Shelf Life)	Products	Lot #	Expiration Date	Storage Time	MW per FFF-MALS	Mean MW	Diff. from 3483*	Aggregation Found (Yes/No)
At Release	AMP- Glucagon	102017	Sept. 2020	1 month	3748	3612	3.7%	No
		102017A		1 month	3550			No
		102017B		1 month	3538			No
	ELI- Glucagon	C699511C	Dec. 2018	11 months	3675	3636	4.4%	No
		C734350C	Feb. 2019	9 months	3666			No
		C753564A	March 2019	8 months	3566			No
At Expiration	AMP-Glucagon	021914	Feb. 2016	45 months	3626	3614	3.8%	No
		021914A		45 months	3713			No
		021914B		45 months	3504			No
	ELI- Glucagon	C559547A	Jan. 2018	22 months	3409	3622	4.0%	No
		C561643C	Jan. 2018	22 months	3738			No
		C561643D	Jan. 2018	22 months	3720			No

* The theoretical molecular weight (MW) of glucagon is 3483 Da.

The molecular weight of the bovine serum albumin (BSA) standard sample was assessed to validate the accuracy of the system. The BSA sample was injected in the same sequence with glucagon samples. The calculated MW of all BSA samples and the percentage of deviation from theoretical MW of BSA (66.5 kDa) are summarized in Table 6. The calculated MWs of all BSA samples were within a 10% deviation from the theoretical value. The results demonstrate that FFF-MALS is an effective method in providing accurate molecular weight determinations.

**Table 6:** BSA standard sample MW summary.

Samples	Calculated MW (Da)	Deviation from Theo. MW (66.5k Da)	Pass/Fail
BSA (2 mg/mL)	65.63k	-1.3%	Pass
	70.64k	6.2%	Pass
	65.12k	-2.1%	Pass
	63.67k	-4.3%	Pass
	66.26k	-0.4%	Pass
	65.57k	-1.4%	Pass
	64.75k	-2.6%	Pass

### Determination of detection limit of FFF-MALS

**Fig 8:**
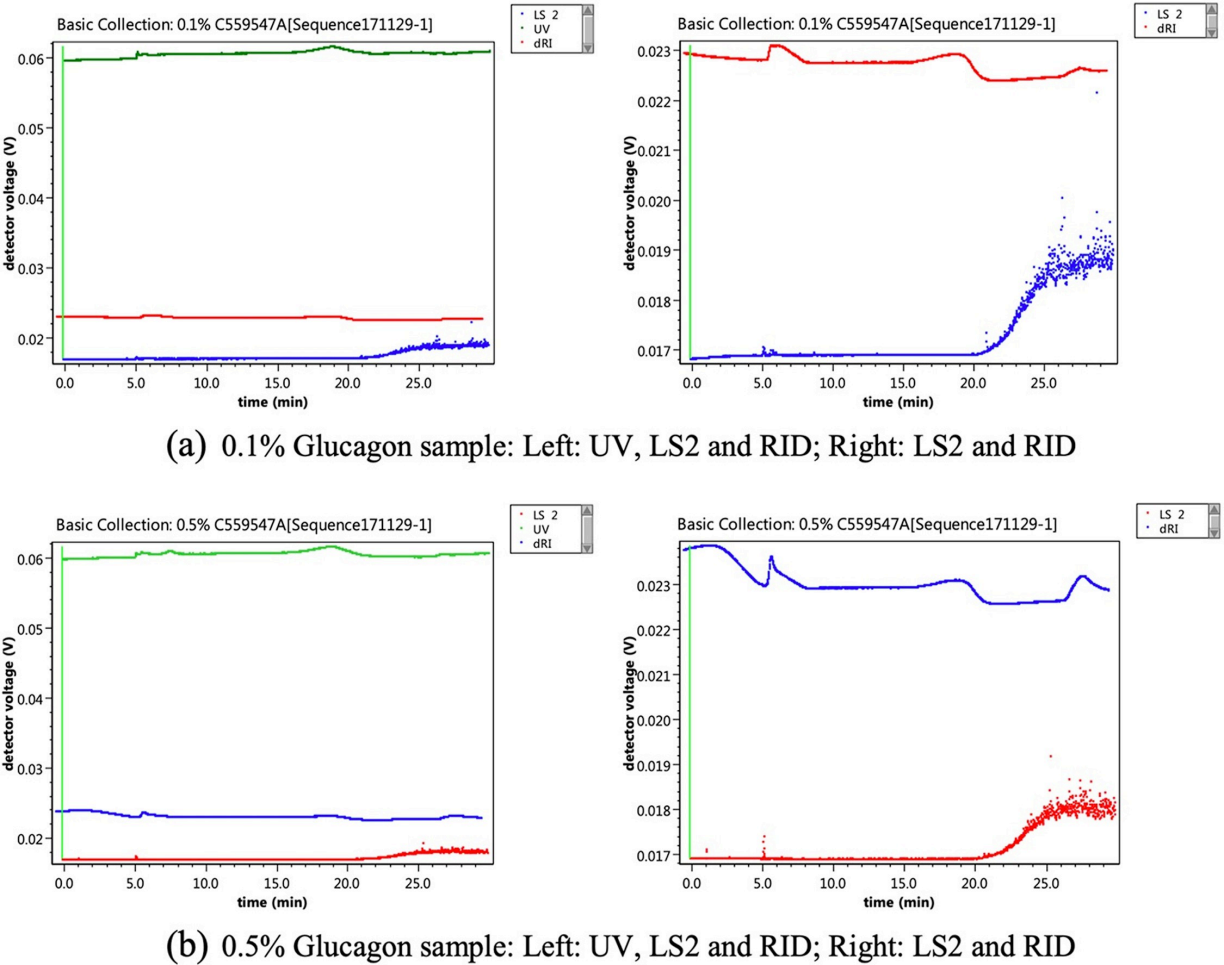
Detection limit of glucagon samples: A reconstituted glucagon sample was diluted to 0.1% and 0.5% of its original concentration. For the 0.1%, the UV, LS2, and RID, no glucagon signal was observed at 7 minutes. For the 0.5%, a very low signal was found at 7 minutes, indicating that the FFF-MALS test is unable to detect glucagon at contents lower than 0.5%.

A reconstituted glucagon sample (ELI-Glucagon Lot C559547A) was used to evaluate the detection limit of the FFF-MALS. The glucagon sample was diluted to 0.1% and 0.5% of its original concentration. The UV, LS2 (90°), and RID responses are shown in Fig 8.

Based on the previous chromatograms in Fig 5, the glucagon monomer peak should appear at around 7 minutes. However, this peak was not detected by all detectors for the 0.1% diluted sample (Fig 8A). For the 0.5% sample (Fig 8B), a very low UV peak appeared at around 7 minutes, but no peaks were detected for the LS2 and RID. This suggests that the FFF-MALS test is not able to detect glucagon aggregates at an amount equal to or lower than 0.5%.

**Fig 9:**
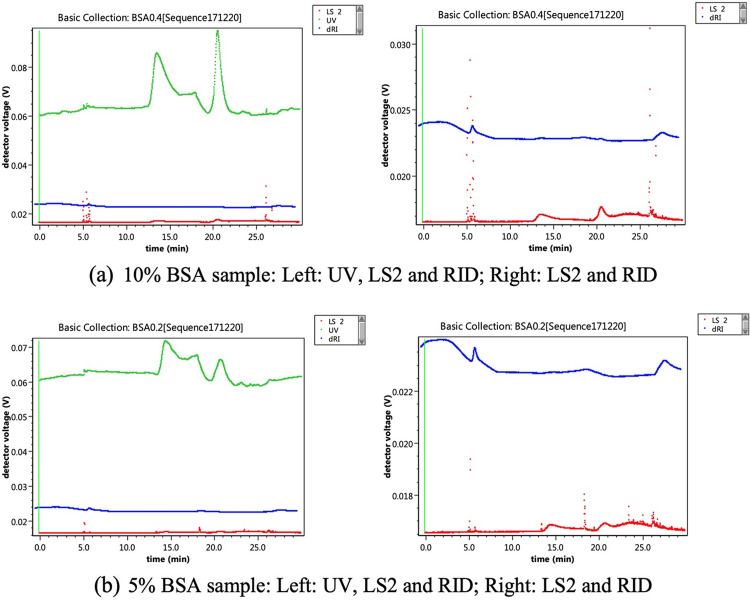
Detection limit of BSA (positive control): The 2 mg/mL BSA sample was diluted to 0.4 and 0.2 mg/mL as positive control samples which is equivalent to 10% and 5% of glucagon sample loaded respectively. The BSA peak should appear at 13–16 minutes based on the UV signals.

A positive control test was also performed using 2 mg/mL BSA sample diluted to 0.4 and 0.2 mg/mL. The diluted samples were equivalent to 10% and 5% of the loaded glucagon samples, respectively. UV, LS2 and RID responses are shown in Fig 9.

The BSA peak should appear at around 13–16 minutes based on the UV signals. For the 5% sample, LS2 and RID signals were found to be very low. The MWs calculated from five injections of this level are 71.54, 78.59, 49.88, 61.19, and 70.60 kDa respectively. The average (66.4 kDa) MW was found to be very similar to that of the theoretical value (66.5 kDa). However, the fluctuations between the five measurements were volatile (i.e. values ranged from 49.88 to 78.59 kDa). Therefore, it is likely that aggregated glucagon that appeared in the drug product was at an amount lower than 5% and was undetected by the FFF-MALS method.

## Discussion

While glucagon is known to be prone to aggregation in solution, both the generic and brand glucagon were quite stable under clinically relevant conditions (i.e. lyophilized powder reconstituted to 1 mg/mL with pH 2.0 diluent and used immediately). A very small amount of dimer (0.1%) was detected by SEC-HPLC in the expired lots of AMP-Glucagon, which is similar to or less than that of the ELI-Glucagon. In the FFF-MALS however, dimer peaks or higher molecular aggregates were not found for either AMP-Glucagon or ELI-Glucagon. An evaluation of the detection limit found that the FFF-MALS method cannot detect aggregated glucagon at amounts lower than 0.5% of total glucagon. These findings were in agreement with the SEC-HPLC results, which determined the dimer contents in AMP-glucagon and ELI-glucagon to be around 0.1%. Assuming this is the actual level of the glucagon dimer in the sample, it is undetectable with the FFF-MALS method.

Since the sensitivity of light scattering increases with increasing molecular weight, the light scattering methods can be effective for detecting small amounts of very large species [19]. For example, in a study using a type of monoclonal antibodies with a large MW of about 150 kDa, dimer contents of 11 to 15% were detected with the FFF application [20]. However, the light scattering method of FFF-MALs is less sensitive in detecting small peptides such as glucagon (MW of 3.5 kDA), despite using the largest injection volume of 100 μL that the Agilent 1100 LC system allows.

More recently, an ELISA method using amyloid fibril-specific antibodies was reported to have a fibril detection limit of ∼0.5–1 ppm in detecting glucagon aggregation [21], which is more sensitive than the detection limit for FFF-MALS method. However, FFF-MALS method allows for a comprehensive characterization of glucagon including monomers and smaller aggregates such as dimers, which is suitable when comparing different glucagon products. Nonetheless, a future research integrating the amyloid fibril-specific antibodies method may be needed to validate the aggregation profile of glucagon products studied in this article.

## Conclusions

Despite the difference in the glucagon origin (synthesis vs rDNA), the generic glucagon (synthetic) was demonstrated to have comparable aggregation profile to that of the brand glucagon (rDNA origin).

## Supporting information

S1 Appendix SEC-HPLC chromatograms for all the study lots. (PDF)

S2 Appendix FFF-MAL chromatograms for all the study lots. (PDF)
