# Supplementary material for: Analysis of aggregation profile of glucagon using SEC-HPLC and FFF-MALS methods
Source: PLoS One. 2024 May 21;19(5):e0304086. doi: 10.1371/journal.pone.0304086 (PMC11108154; doi:10.1371/journal.pone.0304086)
Supplement: S1 Appendix — (PDF) [file pone.0304086.s001.pdf]

S1 Appendix. SEC-HPLC chromatograms for the study glucagon lots:

Study Product	Time Shelf Life	Lot No.
AMP-Glucagon	Recently Released	102017
		102017A
		102017B
	End of Shelf Life (expired)	021914
		021914A
		021914B
ELI-Glucagon	Recently Released	C734350C
		C699511C
		C753564A
	End of Shelf Life (expired)	C502399D
		C505143A
		C464099D

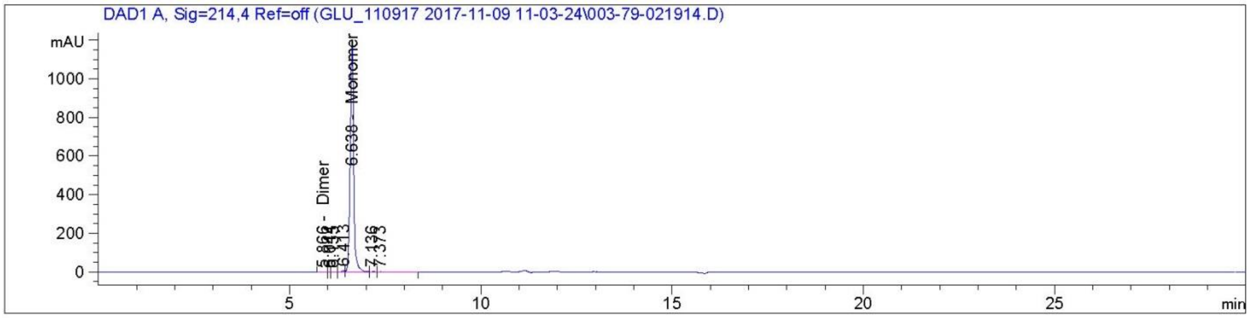
DAD1 A, Sig=214,4 Ref=off (GLU_110917 2017-11-09 11-03-24\003-79-021914.D)
mAU
1000
800
600
400
200
0
5.866 - Dimer
6.638 - Monomer
6.413
7.136
7.373
5
10
15
20
25
min

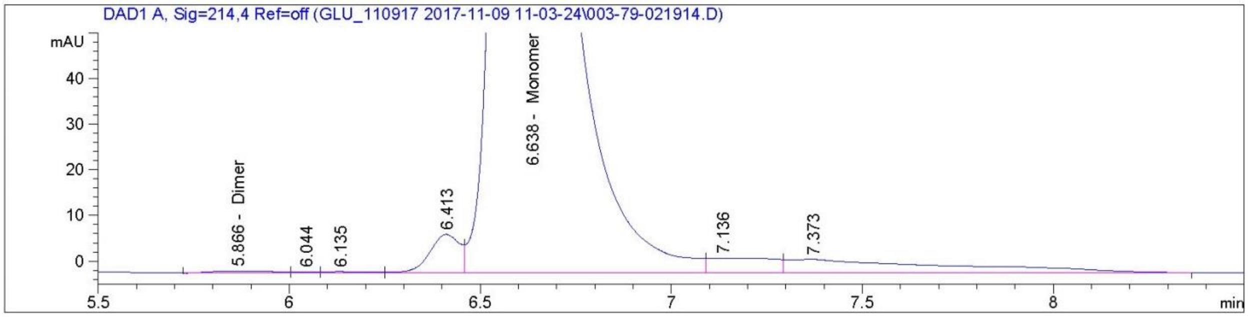
DAD1 A, Sig=214,4 Ref=off (GLU_110917 2017-11-09 11-03-24\003-79-021914.D)
mAU
40
30
20
10
0
5.866 - Dimer
6.044
6.135
6.413
6.638 - Monomer
7.136
7.373
5.5
6
6.5
7
7.5
8
min

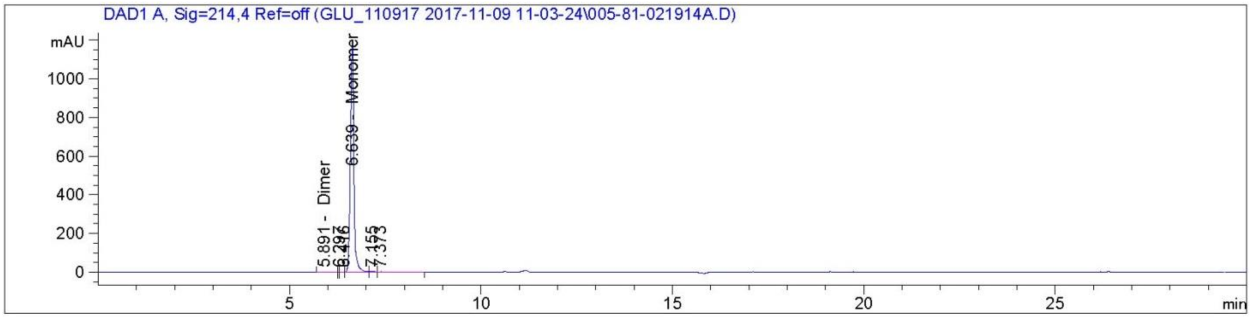
DAD1 A, Sig=214,4 Ref=off (GLU_110917 2017-11-09 11-03-24\005-81-021914A.D)
mAU
1000
800
600
400
200
0
5.891 - Dimer
6.639 - Monomer
7.155
7.373
5
10
15
20
25
min

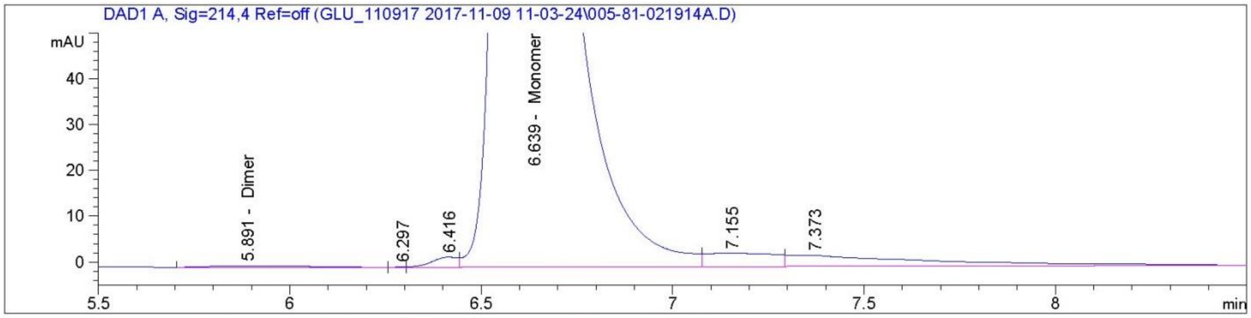
DAD1 A, Sig=214,4 Ref=off (GLU_110917 2017-11-09 11-03-24\005-81-021914A.D)
mAU
40
30
20
10
0
5.891 - Dimer
6.297
6.416
6.639 - Monomer
7.155
7.373
5.5
6
6.5
7
7.5
8
min

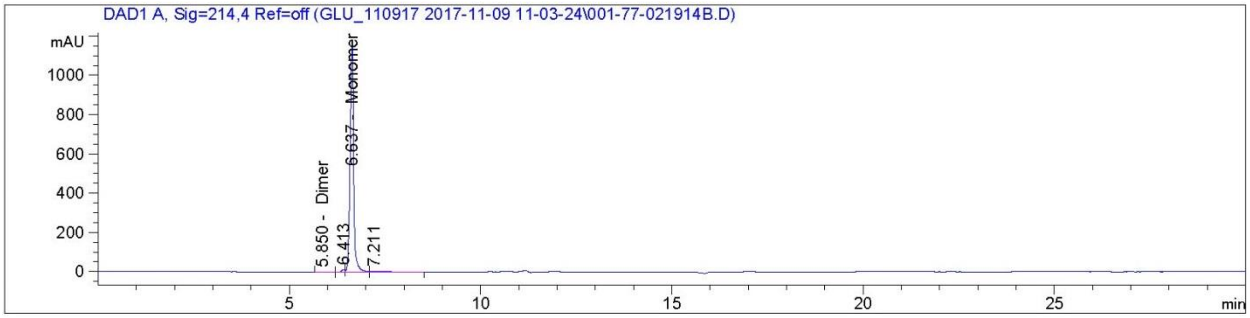
DAD1 A, Sig=214,4 Ref=off (GLU_110917 2017-11-09 11-03-24\001-77-021914B.D)
mAU
1000
800
600
400
200
0
5.850 - Dimer
6.413
6.637 - Monomer
7.211
5
10
15
20
25
min

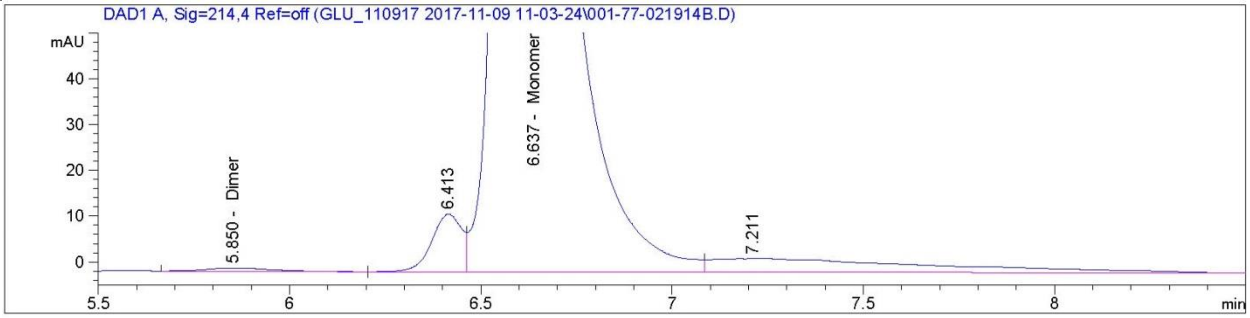
DAD1 A, Sig=214,4 Ref=off (GLU_110917 2017-11-09 11-03-24\001-77-021914B.D)
mAU
5.850 - Dimer
6.413
6.637 - Monomer
7.211
min

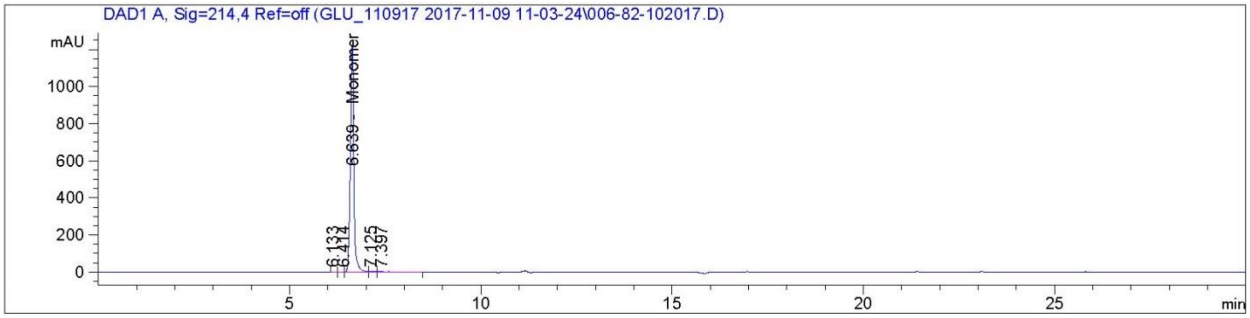
DAD1 A, Sig=214,4 Ref=off (GLU_110917 2017-11-09 11-03-24\006-82-102017.D)
mAU
6.639 - Monomer
6.133
6.414
7.125
7.397
min

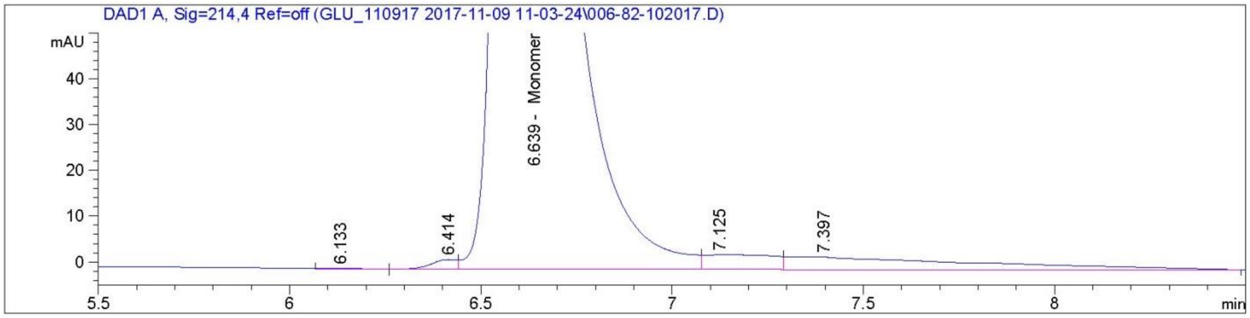
DAD1 A, Sig=214,4 Ref=off (GLU_110917 2017-11-09 11-03-24\006-82-102017.D)
mAU
6.133
6.414
6.639 - Monomer
7.125
7.397
min

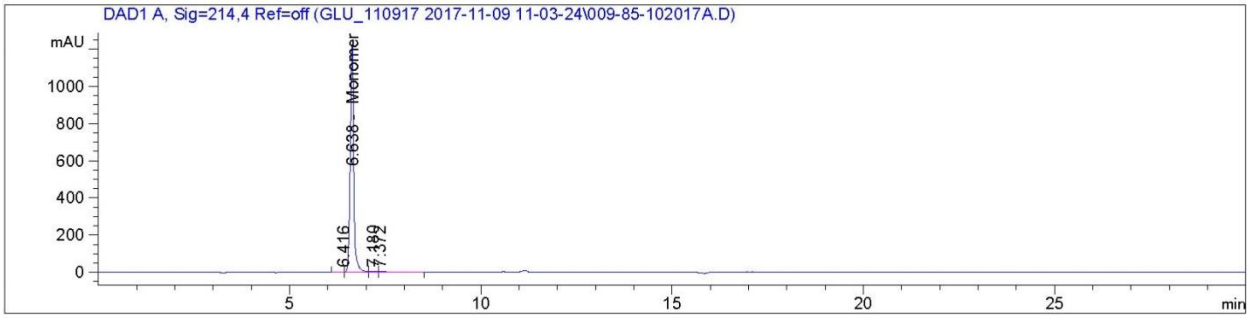
DAD1 A, Sig=214,4 Ref=off (GLU_110917 2017-11-09 11-03-24\009-85-102017A.D)
mAU
6.638 - Monomer
6.416
7.372
min

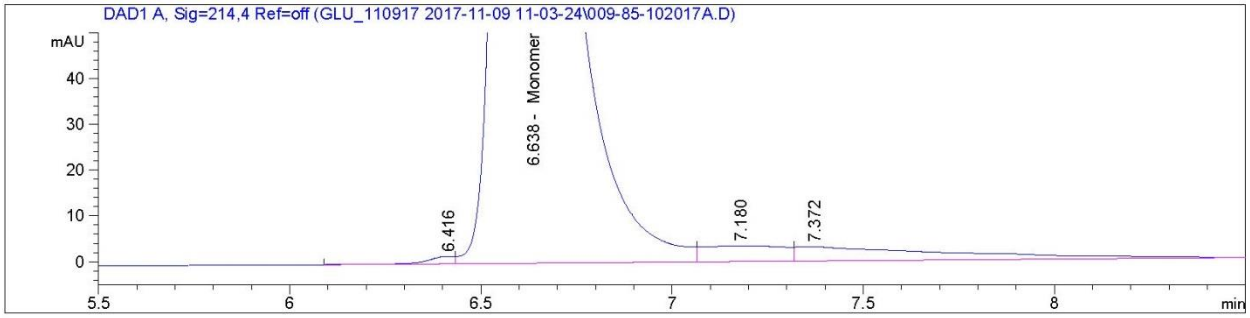
DAD1 A, Sig=214,4 Ref=off (GLU_110917 2017-11-09 11-03-24\009-85-102017A.D)
mAU
6.416
6.638 - Monomer
7.180
7.372
min

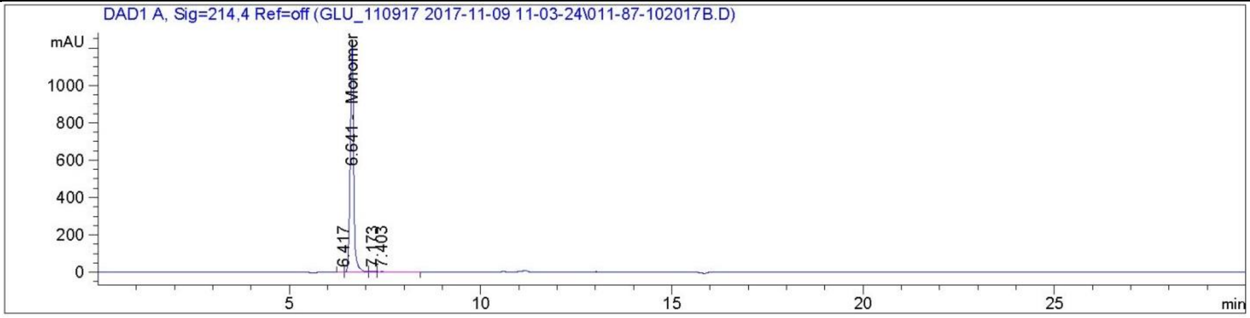
DAD1 A, Sig=214,4 Ref=off (GLU_110917 2017-11-09 11-03-24\011-87-102017B.D)
mAU
6.641 - Monomer
6.417
7.173
7.403
min

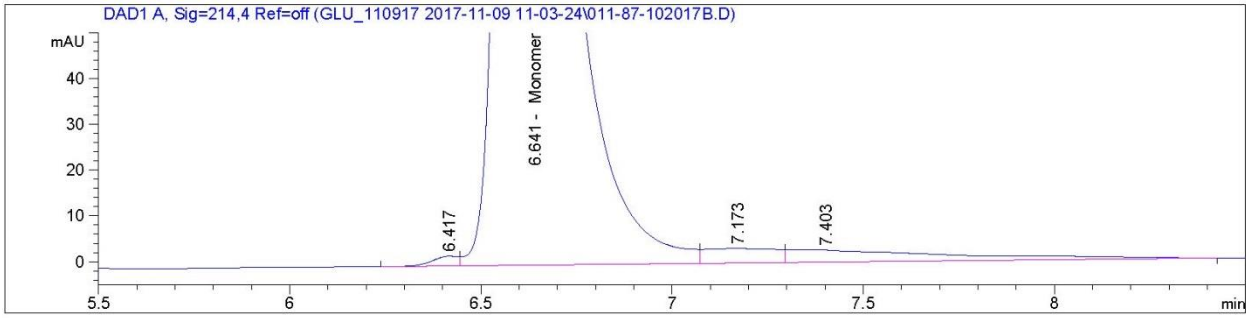
DAD1 A, Sig=214,4 Ref=off (GLU_110917 2017-11-09 11-03-24\011-87-102017B.D)
mAU
6.641 - Monomer
6.417
7.173
7.403
min

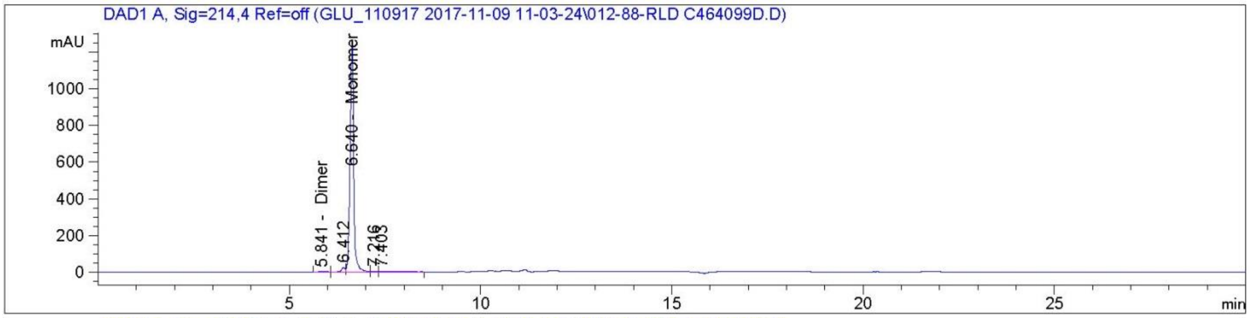
DAD1 A, Sig=214,4 Ref=off (GLU_110917 2017-11-09 11-03-24\012-88-RLD C464099D.D)
mAU
6.640 - Monomer
5.841 - Dimer
6.412
7.216
7.403
min

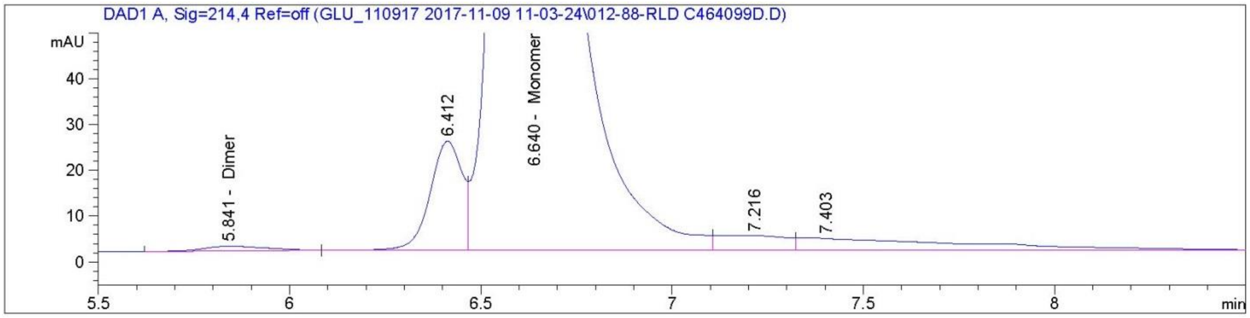
DAD1 A, Sig=214,4 Ref=off (GLU_110917 2017-11-09 11-03-24\012-88-RLD C464099D.D)
mAU
5.841 - Dimer
6.412
6.640 - Monomer
7.216
7.403
min

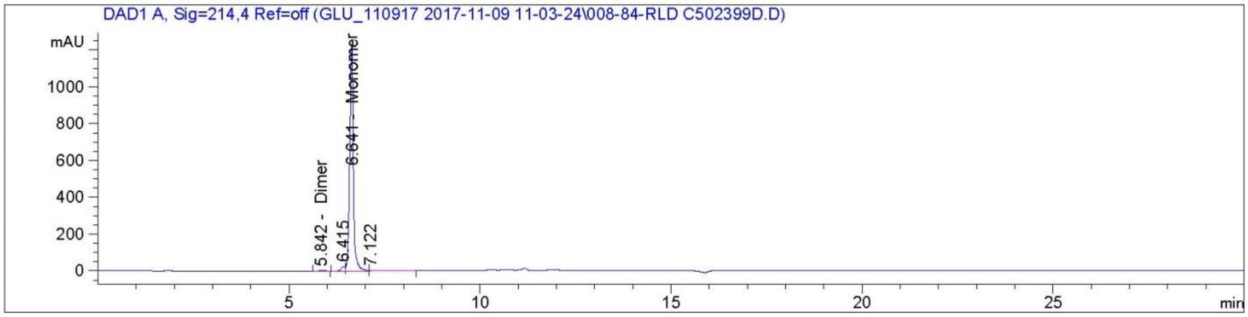
DAD1 A, Sig=214,4 Ref=off (GLU_110917 2017-11-09 11-03-24\008-84-RLD C502399D.D)
mAU
6.641 - Monomer
5.842 - Dimer
6.415
7.122
min

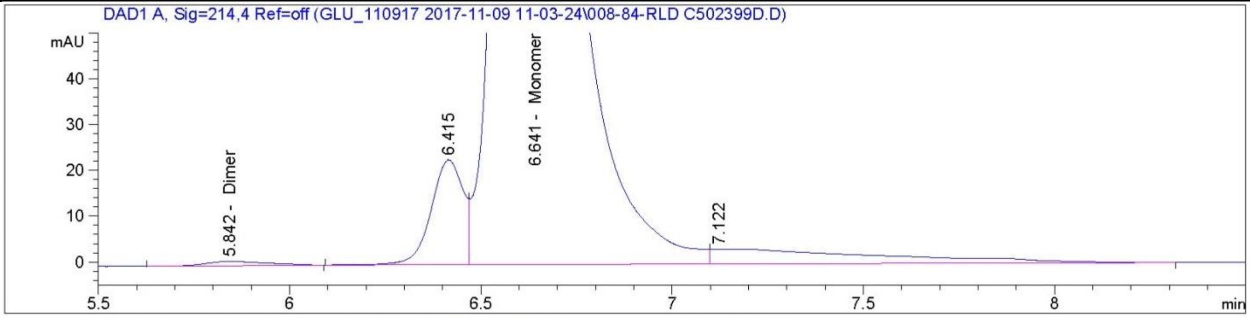
DAD1 A, Sig=214,4 Ref=off (GLU_110917 2017-11-09 11-03-24\008-84-RLD C502399D.D)
mAU
5.842 - Dimer
6.415
6.641 - Monomer
7.122
min

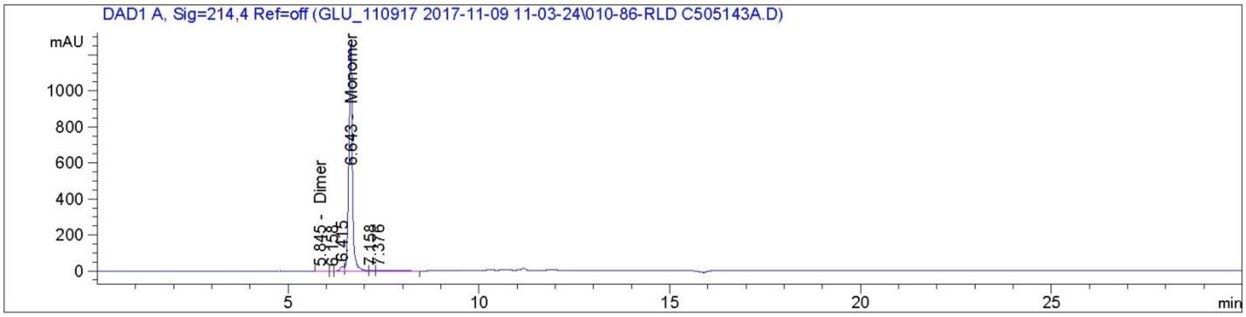
DAD1 A, Sig=214,4 Ref=off (GLU_110917 2017-11-09 11-03-24\010-86-RLD C505143A.D)
mAU
5.845 - Dimer
6.643 - Monomer
min

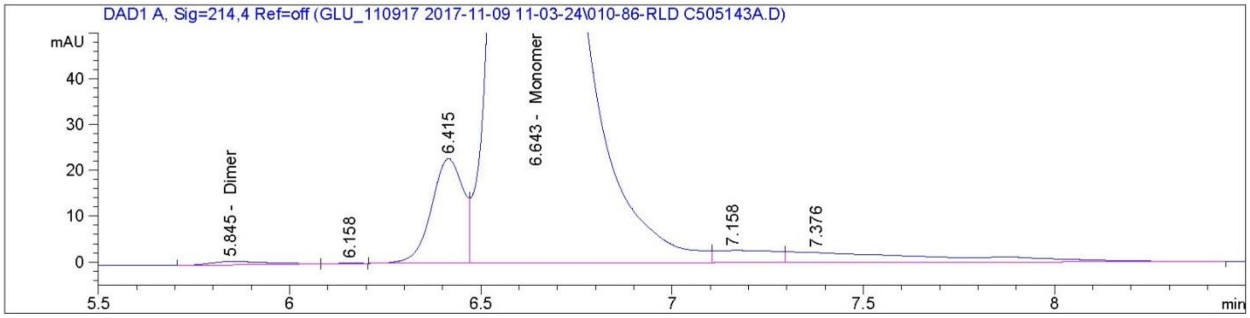
DAD1 A, Sig=214,4 Ref=off (GLU_110917 2017-11-09 11-03-24\010-86-RLD C505143A.D)
mAU
5.845 - Dimer
6.158
6.415
6.643 - Monomer
7.158
7.376
min

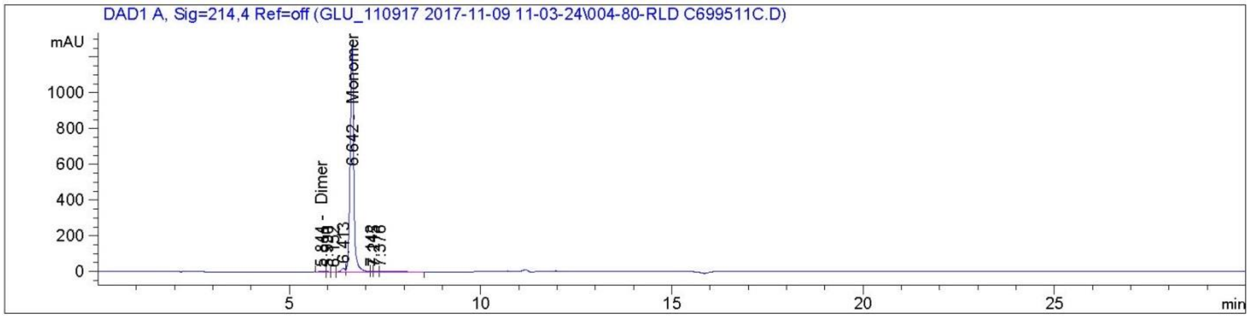
DAD1 A, Sig=214,4 Ref=off (GLU_110917 2017-11-09 11-03-24\004-80-RLD C699511C.D)
mAU
Dimer
6.413
6.642 - Monomer
7.376
min

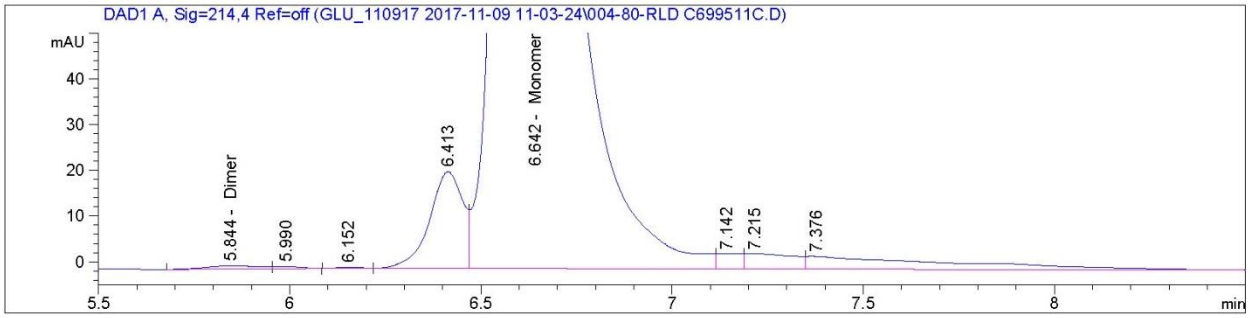
DAD1 A, Sig=214,4 Ref=off (GLU_110917 2017-11-09 11-03-24\004-80-RLD C699511C.D)
mAU
5.844 - Dimer
5.990
6.152
6.413
6.642 - Monomer
7.142
7.215
7.376
min

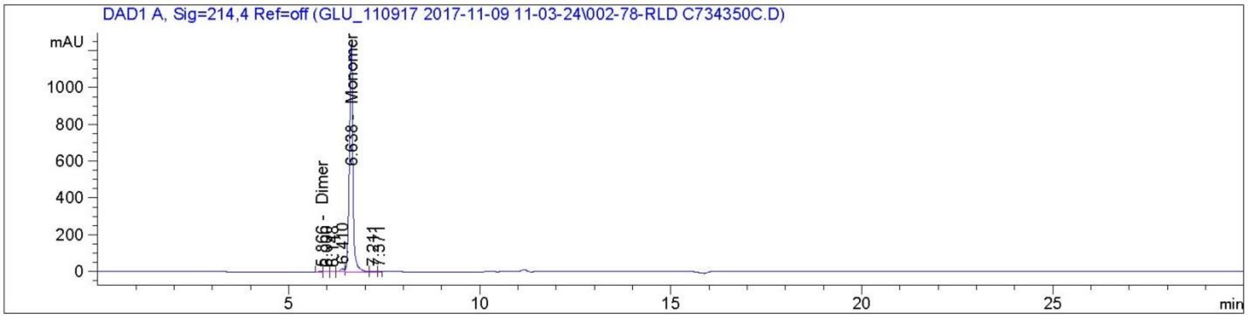
DAD1 A, Sig=214,4 Ref=off (GLU_110917 2017-11-09 11-03-24\002-78-RLD C734350C.D)
mAU
6.638 - Monomer
Dimer
min

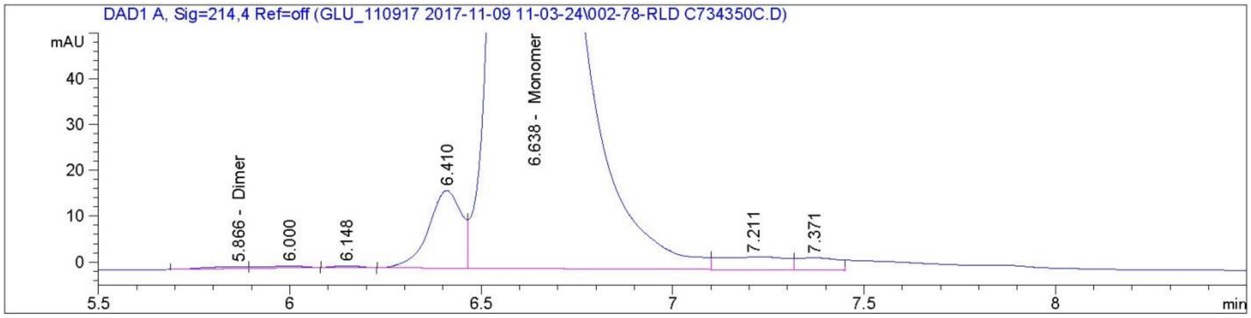
DAD1 A, Sig=214,4 Ref=off (GLU_110917 2017-11-09 11-03-24\002-78-RLD C734350C.D)
mAU
5.866 - Dimer
6.000
6.148
6.410
6.638 - Monomer
7.211
7.371
min

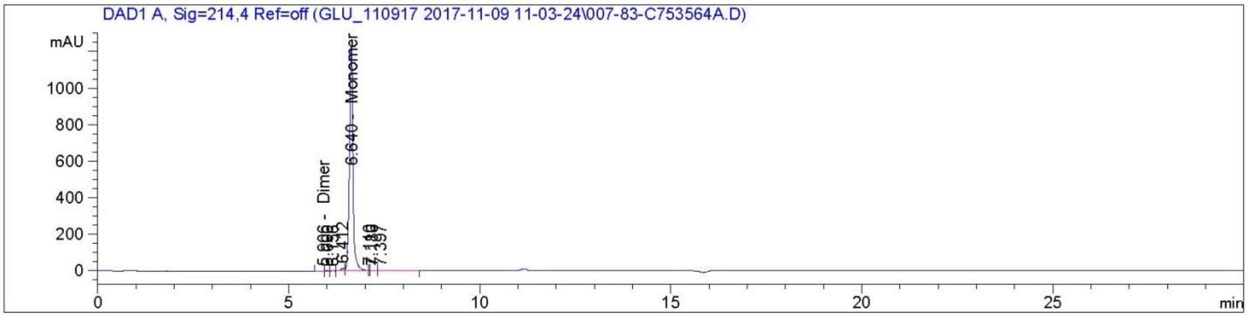
DAD1 A, Sig=214,4 Ref=off (GLU_110917 2017-11-09 11-03-24\007-83-C753564A.D)
mAU
6.640 - Monomer
Dimer
min

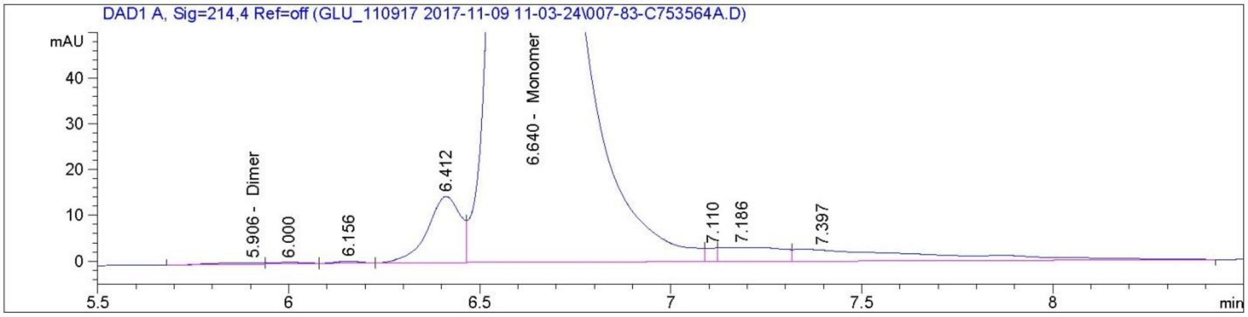
DAD1 A, Sig=214,4 Ref=off (GLU_110917 2017-11-09 11-03-24\007-83-C753564A.D)
mAU
5.906 - Dimer
6.000
6.156
6.412
6.640 - Monomer
7.110
7.186
7.397
min
