# Supplementary material for: Analysis of aggregation profile of glucagon using SEC-HPLC and FFF-MALS methods
Source: PLoS One. 2024 May 21;19(5):e0304086. doi: 10.1371/journal.pone.0304086 (PMC11108154; doi:10.1371/journal.pone.0304086)

S2 Appendix. FFF-MAL chromatograms for the study glucagon lots:

Study Product	Time Shelf Life	Lot No.
AMP-Glucagon	Recently Released	102017
		102017A
		102017B
	End of Shelf Life (expired)	021914
		021914A
		021914B
ELI-Glucagon	Recently Released	C734350C
		C699511C
		C753564A
	End of Shelf Life (expired)	C559547A
		C561643C
		C561643D

Molar Mass vs. time

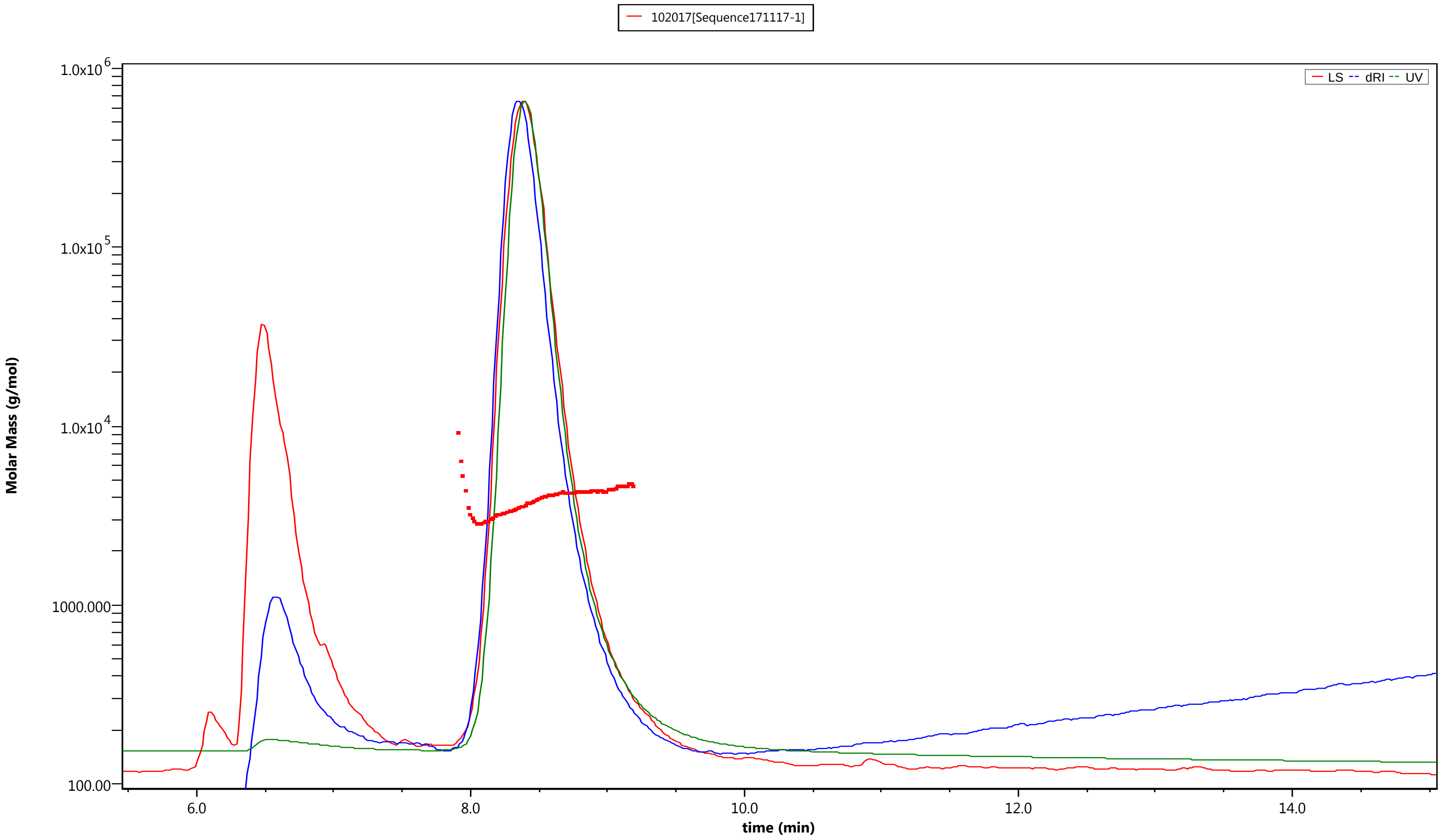

EASI Graph

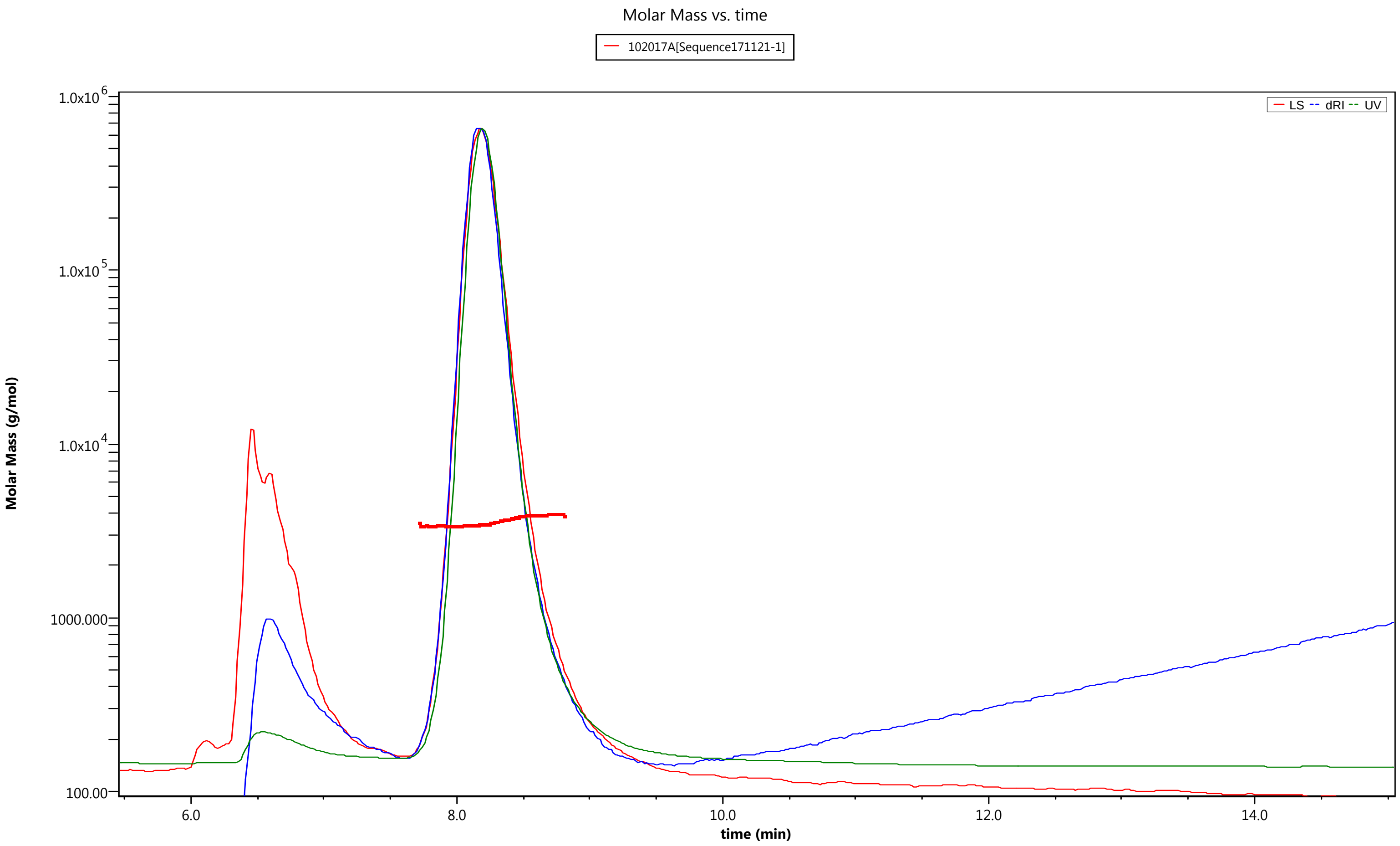

Molar Mass vs. time
102017A[Sequence171121-1]
LS
dRI
UV
1.0x10^6
1.0x10^5
1.0x10^4
1000.000
100.00
Molar Mass (g/mol)
6.0
8.0
10.0
12.0
14.0
time (min)

EASI Graph

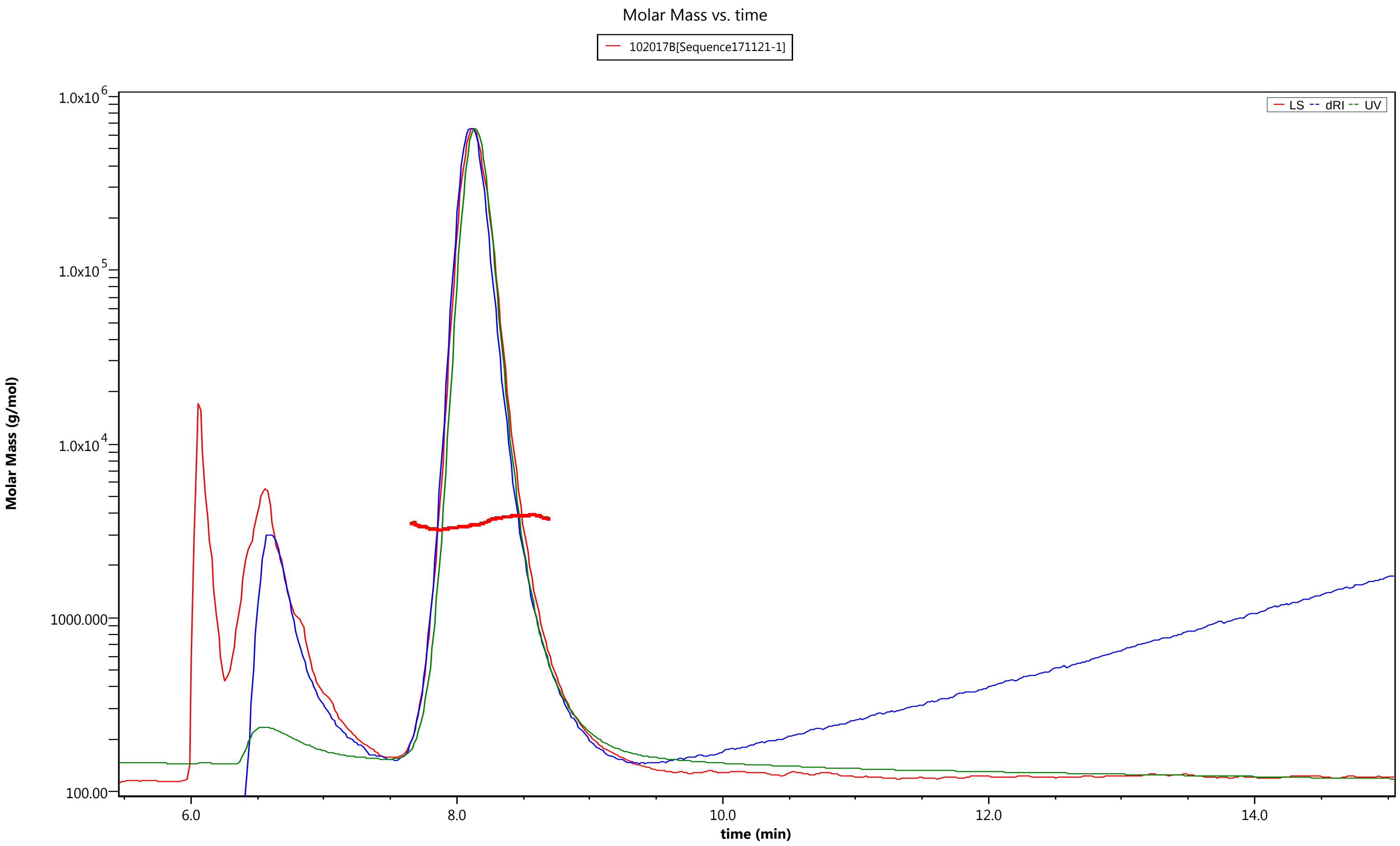

Molar Mass vs. time
102017B[Sequence171121-1]
LS
dRI
UV
Molar Mass (g/mol)
1.0x10^6
1.0x10^5
1.0x10^4
1000.000
100.00
6.0
8.0
10.0
12.0
14.0
time (min)

EASI Graph

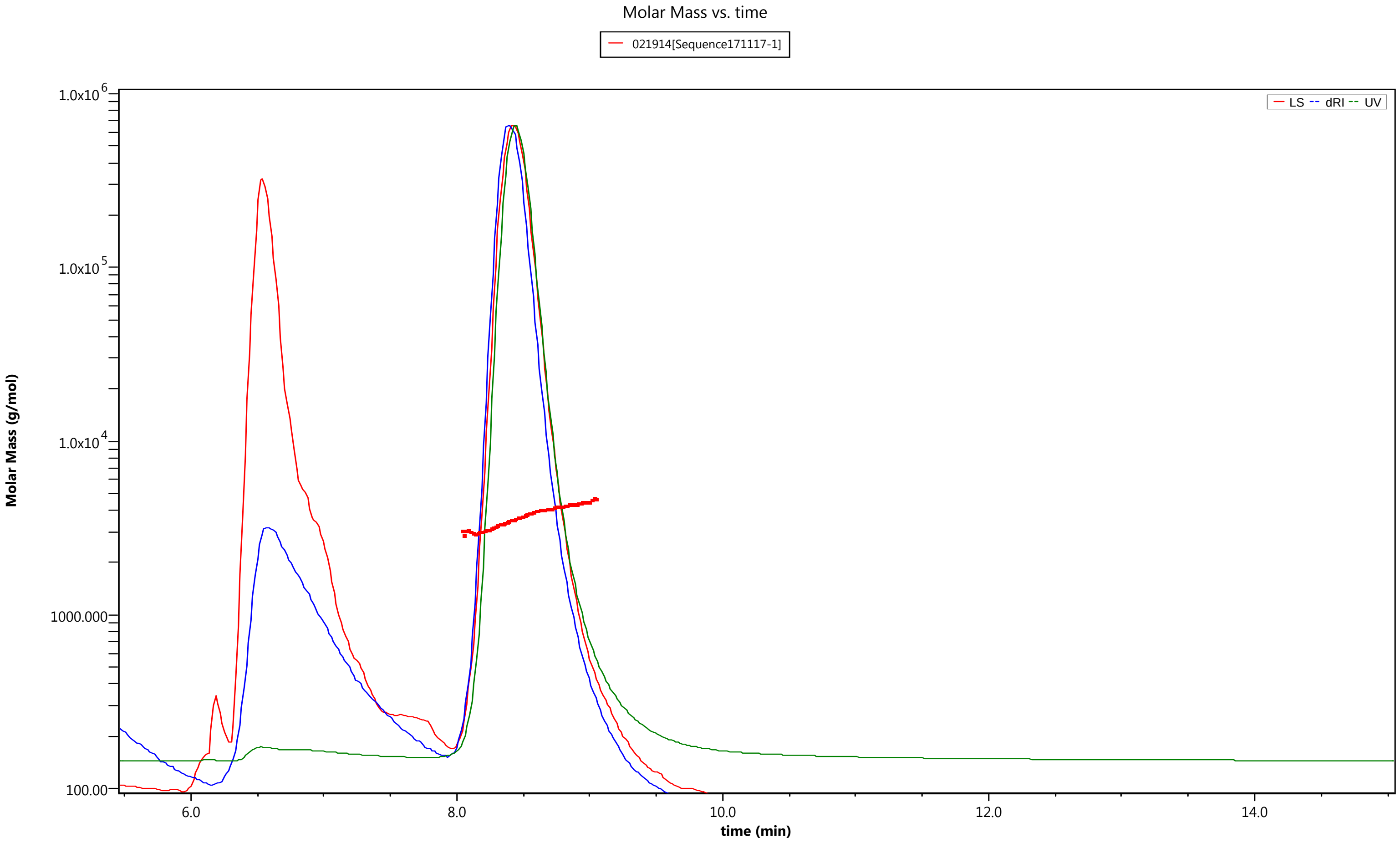

Molar Mass vs. time
021914[Sequence171117-1]
LS
dRI
UV
Molar Mass (g/mol)
1.0x10^6
1.0x10^5
1.0x10^4
1000.000
100.00
6.0
8.0
10.0
12.0
14.0
time (min)

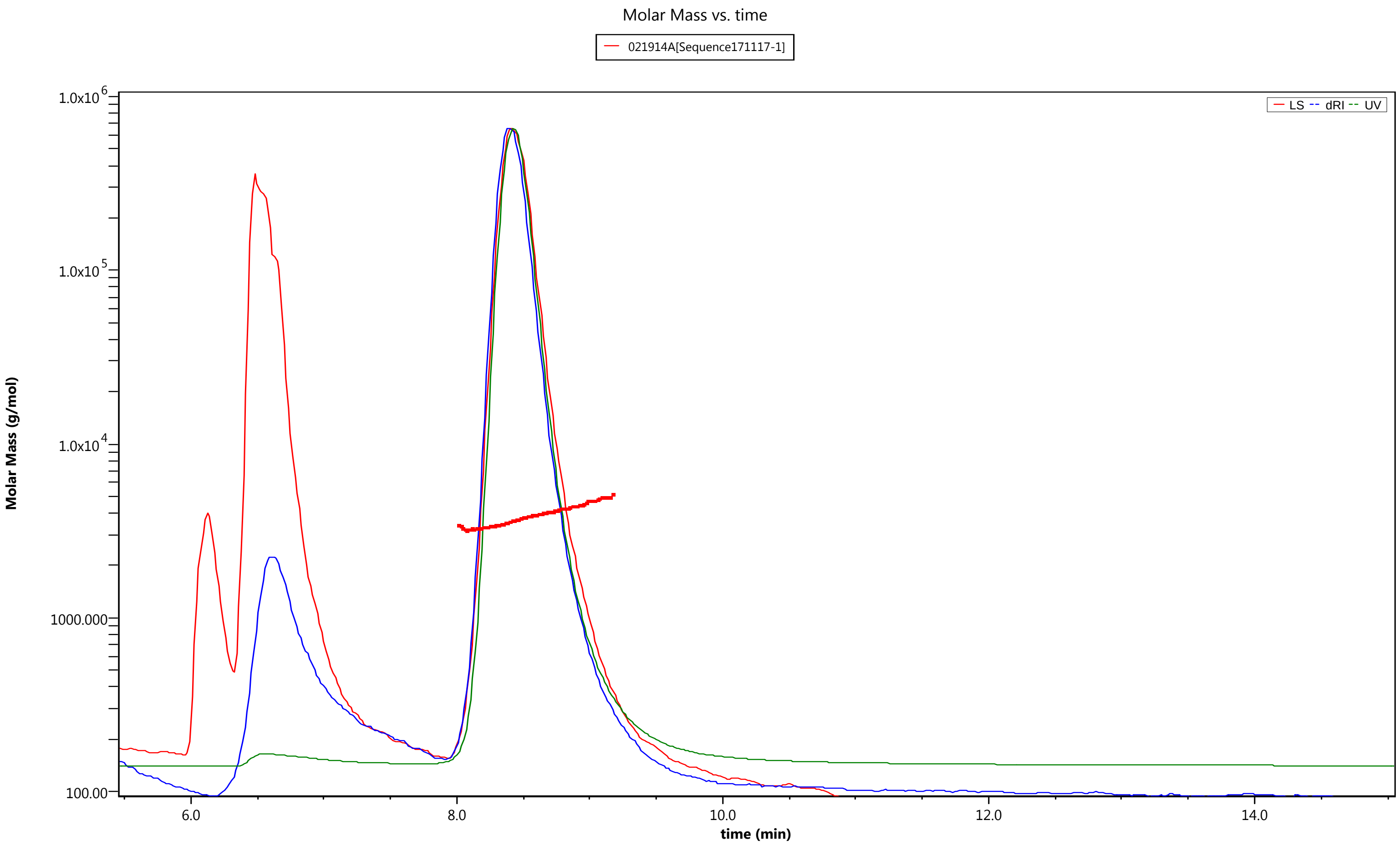

Molar Mass vs. time
021914A[Sequence171117-1]
LS
dRI
UV
Molar Mass (g/mol)
1.0x10^6
1.0x10^5
1.0x10^4
1000.000
100.00
6.0
8.0
10.0
12.0
14.0
time (min)

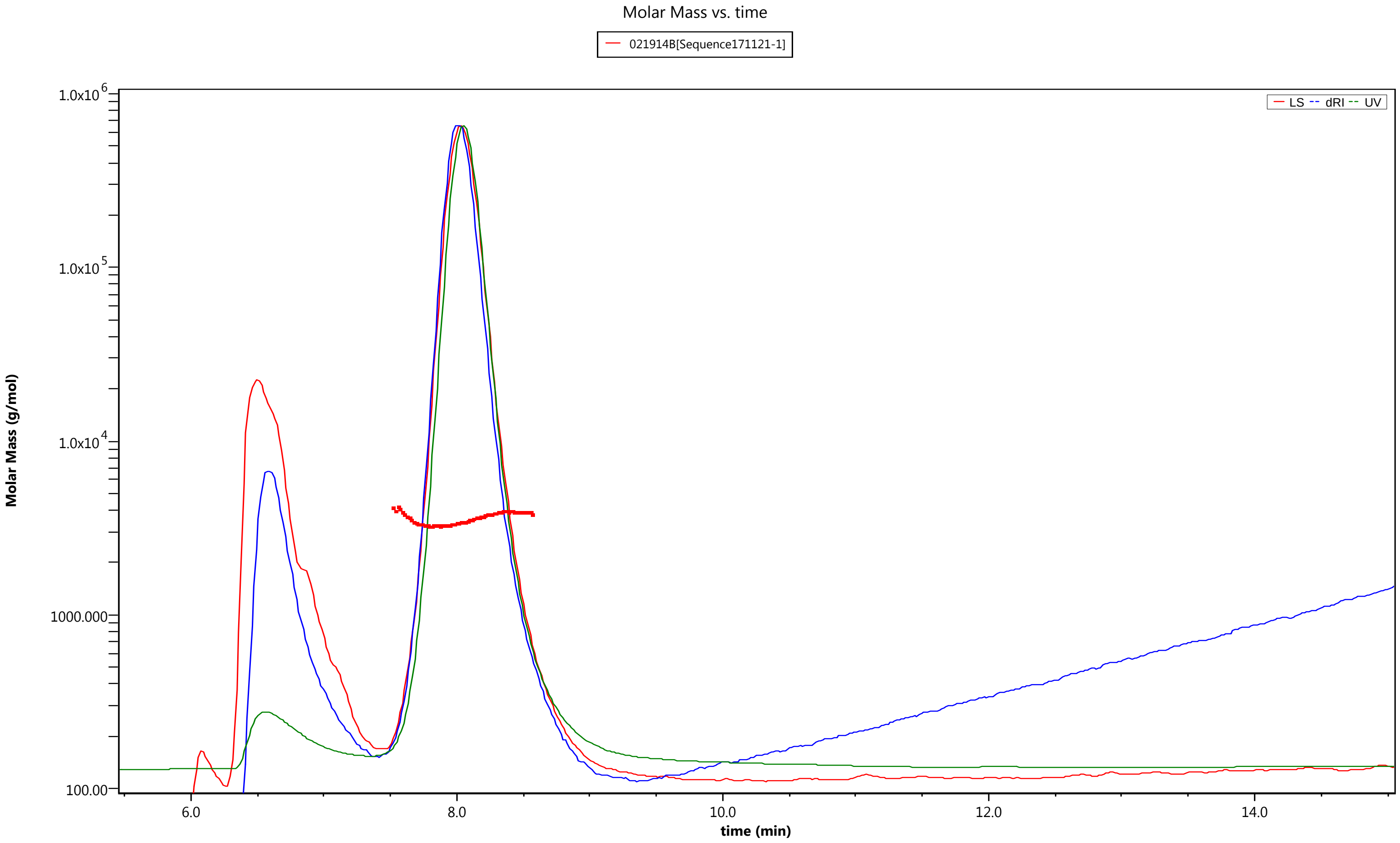

Molar Mass vs. time
021914B[Sequence171121-1]
LS
dRI
UV
Molar Mass (g/mol)
1.0x10^6
1.0x10^5
1.0x10^4
1000.000
100.00
6.0
8.0
10.0
12.0
14.0
time (min)

EASI Graph

Molar Mass vs. time

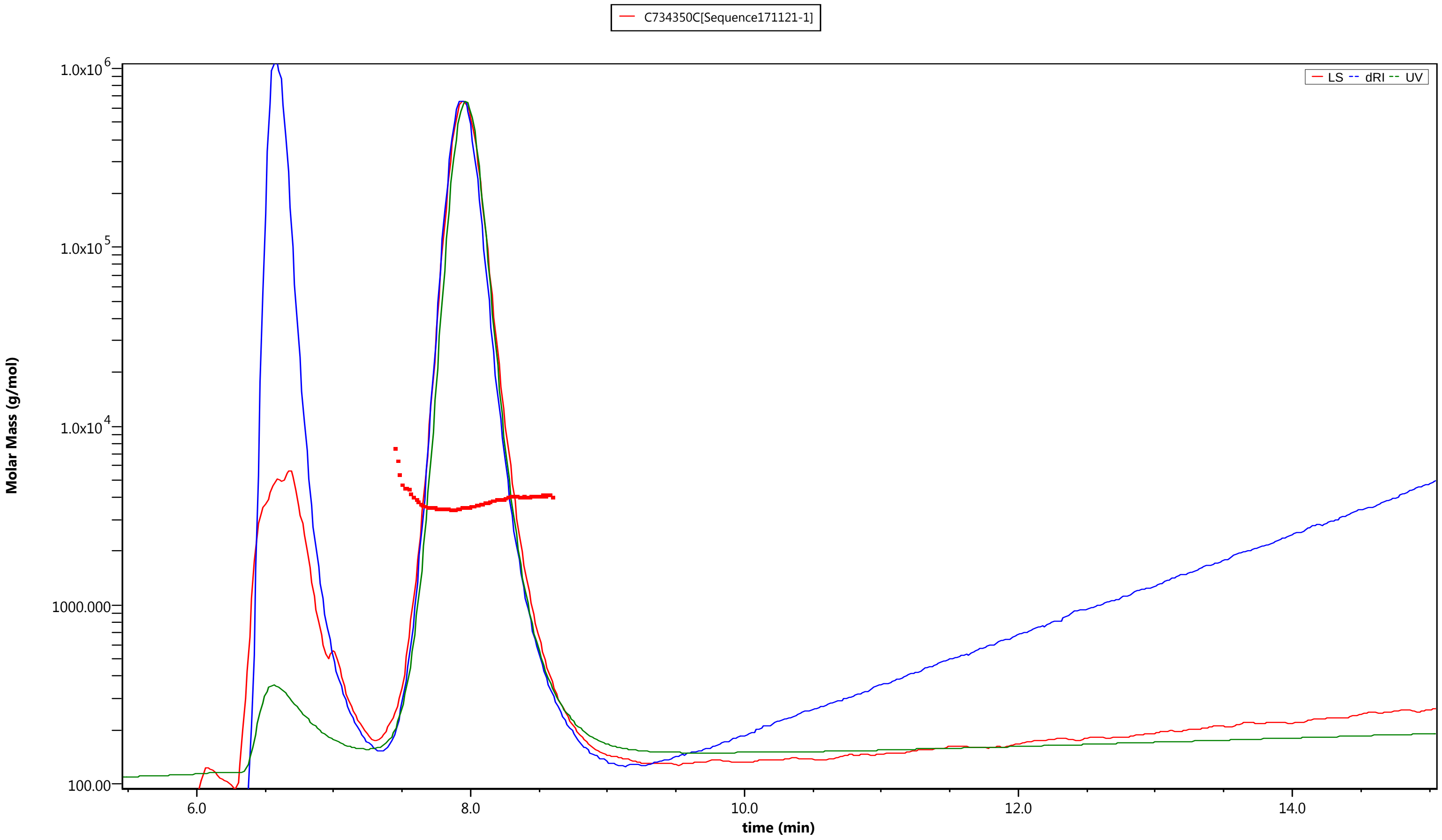

EASI Graph

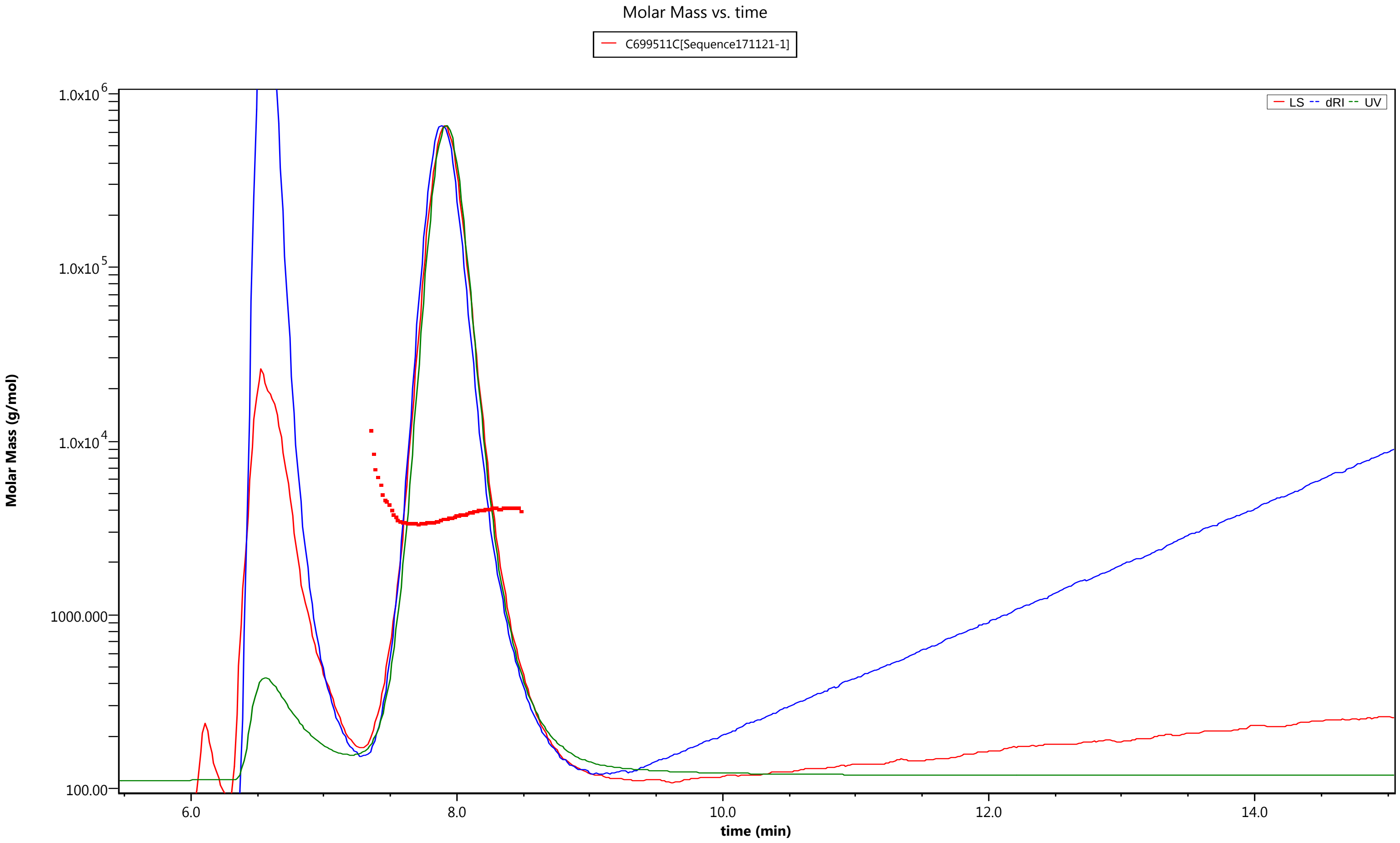

Molar Mass vs. time
C699511C[Sequence171121-1]
LS
dRI
UV
Molar Mass (g/mol)
1.0x10^6
1.0x10^5
1.0x10^4
1000.000
100.00
6.0
8.0
10.0
12.0
14.0
time (min)

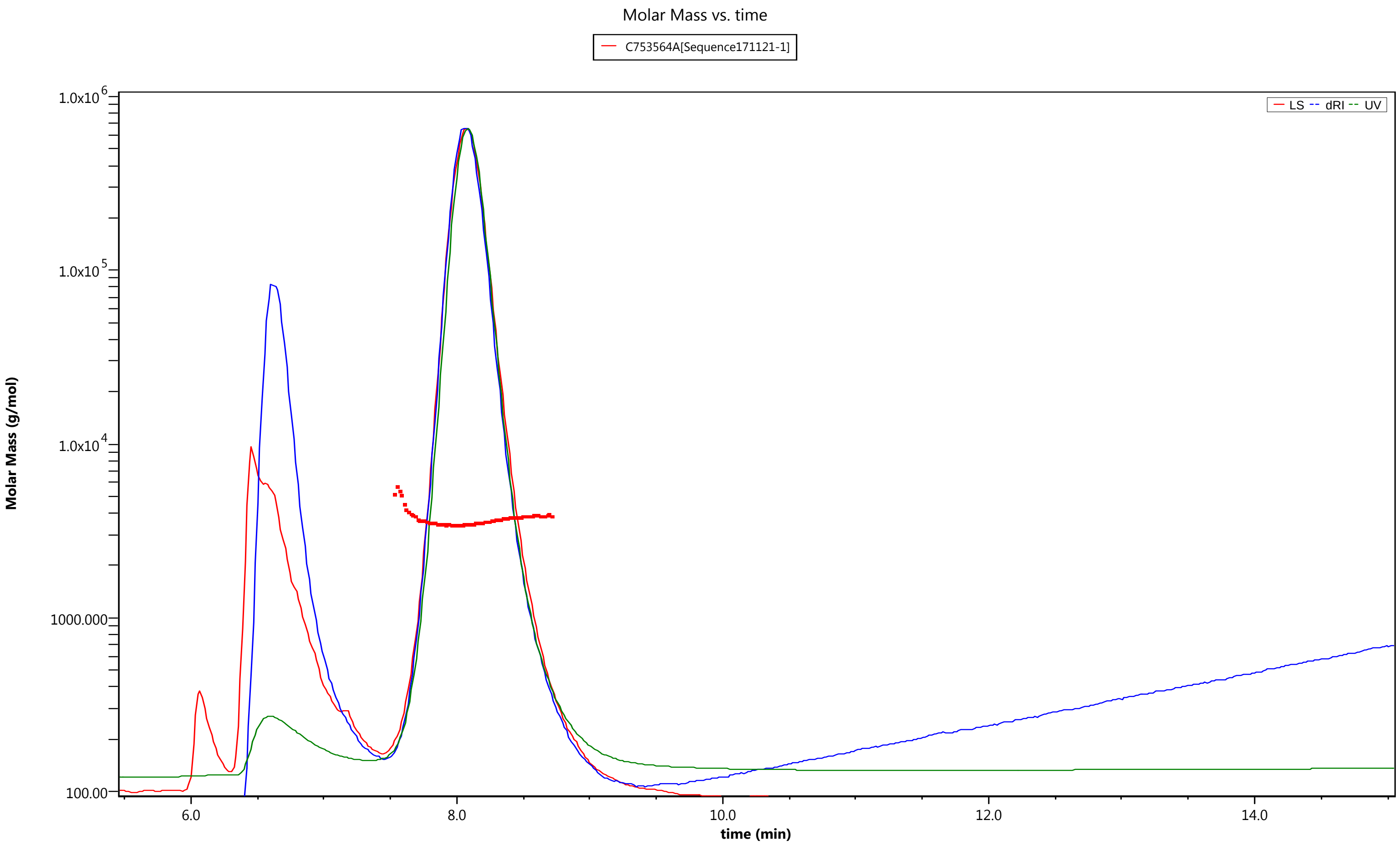
Molar Mass vs. time
C753564A[Sequence171121-1]
LS
dRI
UV
Molar Mass (g/mol)
1.0x10^6
1.0x10^5
1.0x10^4
1000.000
100.00
6.0
8.0
10.0
12.0
14.0
time (min)

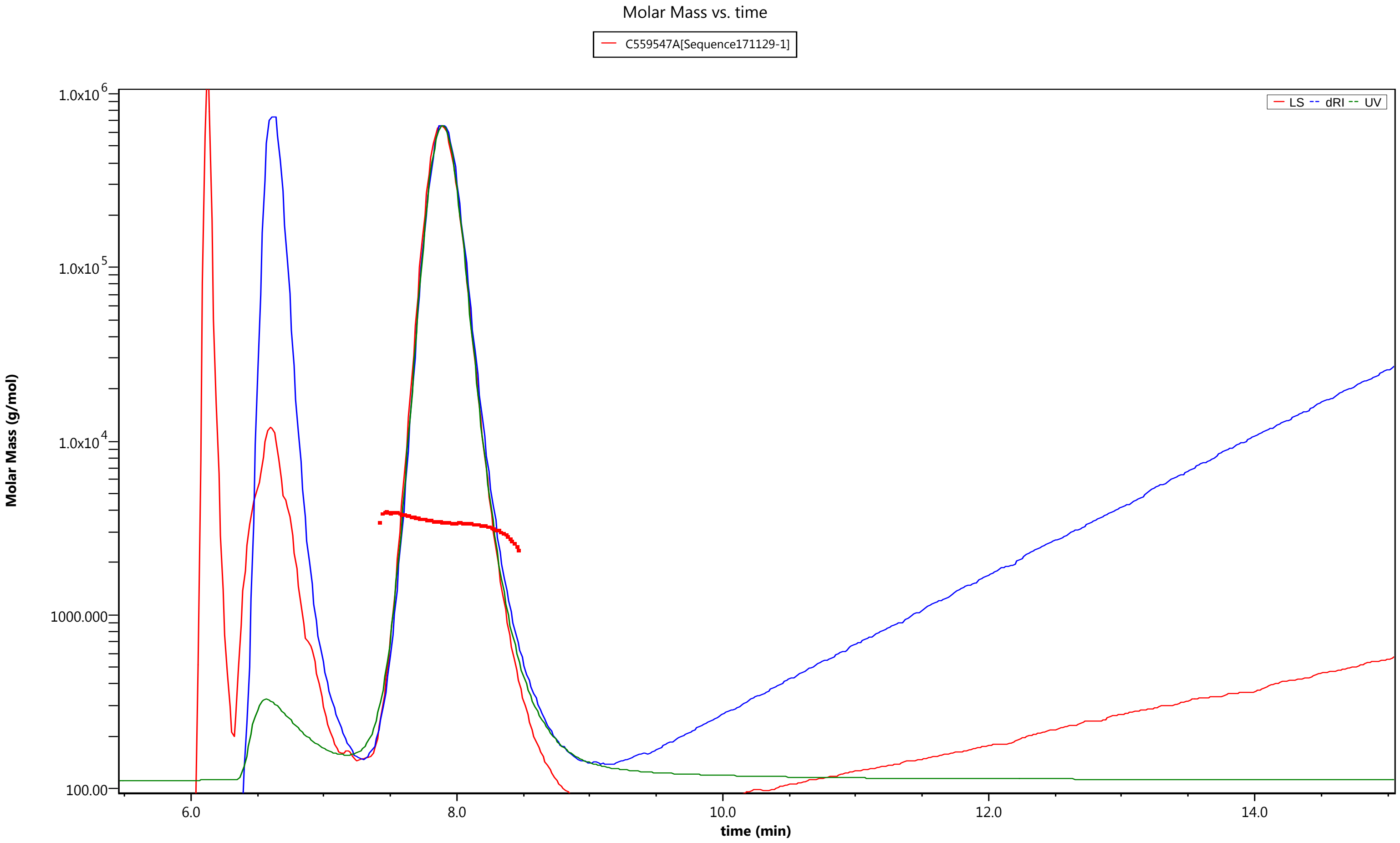

Molar Mass vs. time
C559547A[Sequence171129-1]
LS dRI UV
Molar Mass (g/mol)
1.0x10^6
1.0x10^5
1.0x10^4
1000.000
100.00
6.0
8.0
10.0
12.0
14.0
time (min)

Molar Mass vs. time

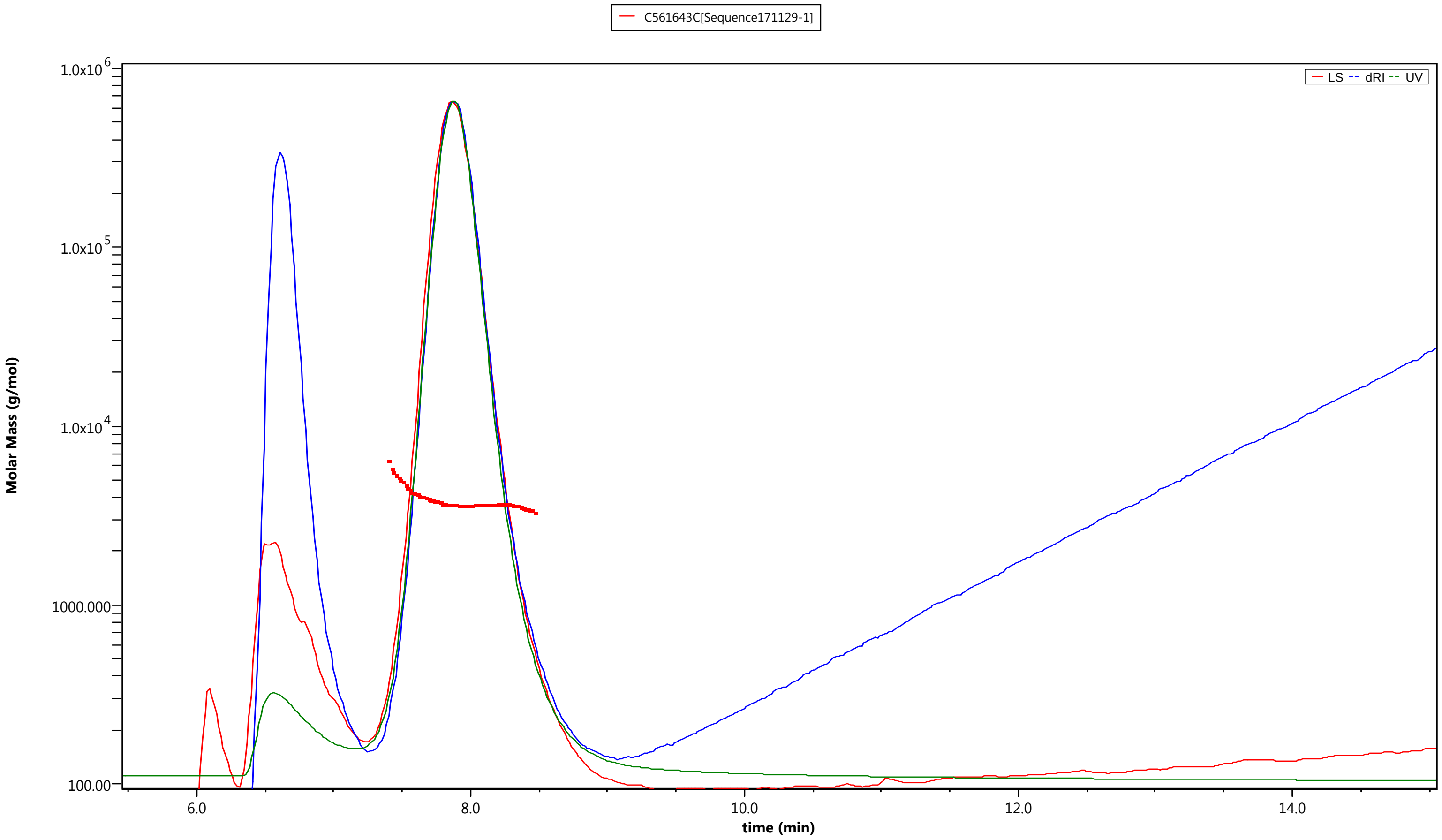

EASI Graph

Molar Mass vs. time

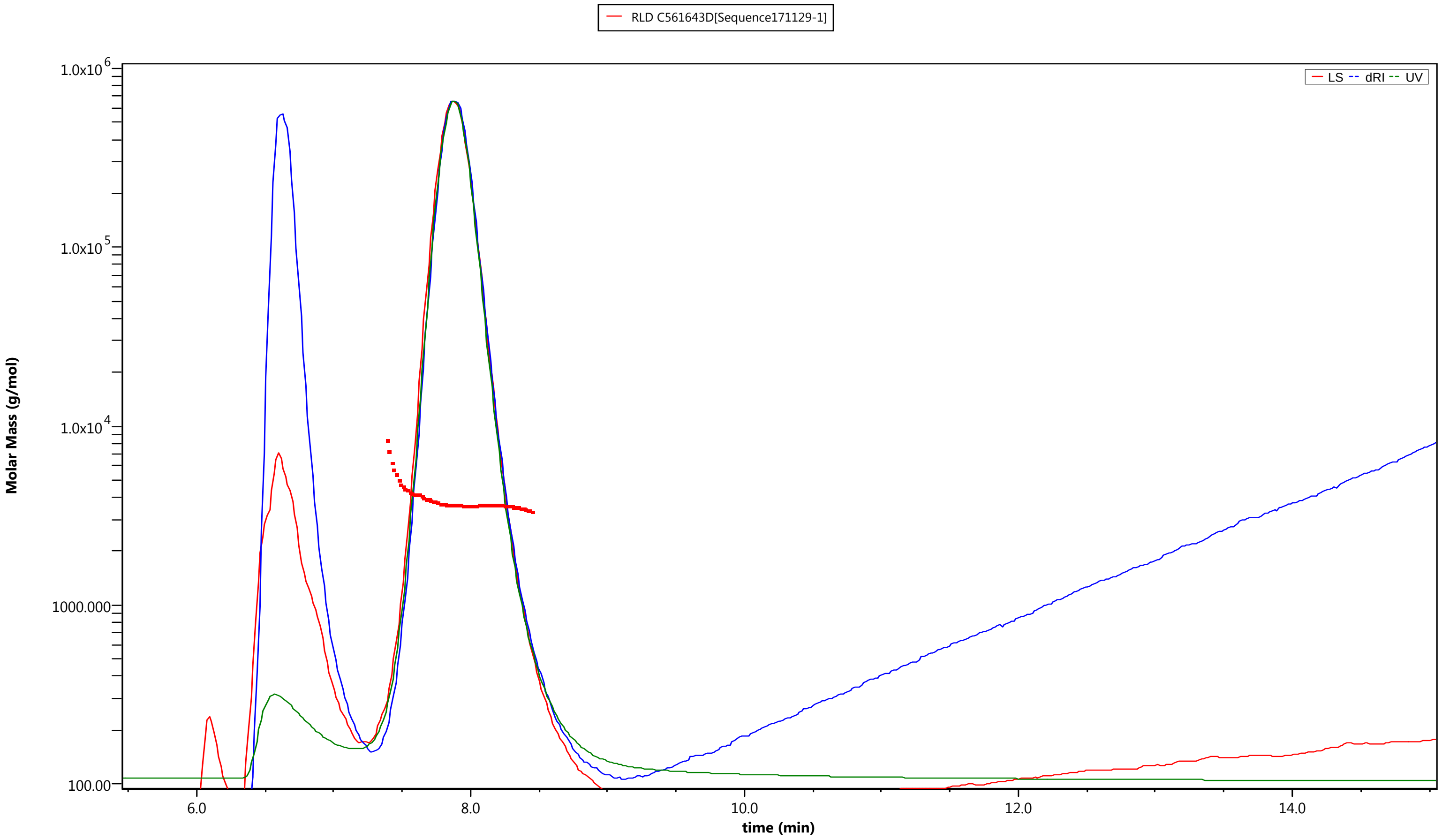

Supplement: S2 Appendix — (PDF) [file pone.0304086.s002.pdf]
